# Inpatient care of small and sick newborns: a multi-country analysis of health system bottlenecks and potential solutions

**DOI:** 10.1186/1471-2393-15-S2-S7

**Published:** 2015-09-11

**Authors:** Sarah G Moxon, Joy E Lawn, Kim E Dickson, Aline Simen-Kapeu, Gagan Gupta, Ashok Deorari, Nalini Singhal, Karen New, Carole Kenner, Vinod Bhutani, Rakesh Kumar, Elizabeth Molyneux, Hannah Blencowe

**Affiliations:** 1 Maternal, Adolescent, Reproductive and Child Health (MARCH) Centre, London School of Hygiene and Tropical Medicine, London, WC1E 7HT, UK; 2 Department of Infectious Disease Epidemiology, London School of Hygiene and Tropical Medicine, London, WC1E 7HT, UK; 3 Saving Newborn Lives, Save the Children, Washington DC, 20036, USA; 4 Health Section, Programme Division, UNICEF Headquarters, 3 United Nations Plaza, New York, 10017, NY, USA; 5 UNICEF, India 73, Lodi Estate New Delhi, 110 003, India; 6 Department of Pediatrics, WHO Collaborating Centre for Education & Research in Newborn Care, All India Institute of Medical Sciences, Ansari Nagar, New Delhi, 110029, India; 7 University of Calgary, 2888, Shaganappi Trail NW, Calgary, Alberta, T3B 6C8, Canada; 8 The University of Queensland, Brisbane, Qld, 4029, Australia; 9 Council of International Neonatal Nurses, Dean of School of Nursing, Health and Exercise Science, The College of New Jersey, Ewing, NJ, 08628-0718, USA; 10 Stanford University School of Medicine, 291 Campus Drive, Li Ka Shing Building, Stanford, CA, 94305-5101, USA; 11 India Ministry of Health & Family Welfare, Government of India, Nirman Bhawan, New Delhi, 110108, India; 12 College of Medicine, Box 360, Blantyre, Malawi

**Keywords:** Sick newborn, preterm, special care, neonatal nurse, inpatient care, facility, bottlenecks, health systems

## Abstract

**Background:**

Preterm birth is the leading cause of child death worldwide. Small and sick newborns require timely, high-quality inpatient care to survive. This includes provision of warmth, feeding support, safe oxygen therapy and effective phototherapy with prevention and treatment of infections. Inpatient care for newborns requires dedicated ward space, staffed by health workers with specialist training and skills. Many of the estimated 2.8 million newborns that die every year do not have access to such specialised care.

**Methods:**

The bottleneck analysis tool was applied in 12 countries in Africa and Asia as part of the *Every Newborn *Action Plan process. Country workshops involved technical experts to complete the survey tool, which is designed to synthesise and grade health system "bottlenecks" (or factors that hinder the scale up) of maternal-newborn intervention packages. For this paper, we used quantitative and qualitative methods to analyse the bottleneck data, and combined these with literature review, to present priority bottlenecks and actions relevant to different health system building blocks for inpatient care of small and sick newborns.

**Results:**

Inpatient care of small and sick newborns is an intervention package highlighted by all country workshop participants as having critical health system challenges. Health system building blocks with the highest graded (significant or major) bottlenecks were health workforce (10 out of 12 countries) and health financing (10 out of 12 countries), followed by community ownership and partnership (9 out of 12 countries). Priority actions based on solution themes for these bottlenecks are discussed.

**Conclusions:**

Whilst major bottlenecks to the scale-up of quality inpatient newborn care are present, effective solutions exist. For all countries included, there is a critical need for a neonatal nursing cadre. Small and sick newborns require increased, sustained funding with specific insurance schemes to cover inpatient care and avoid catastrophic out-of-pocket payments. Core competencies, by level of care, should be defined for monitoring of newborn inpatient care, as with emergency obstetric care. Rather than fatalism that small and sick newborns will die, community interventions need to create demand for accessible, high-quality, family-centred inpatient care, including kangaroo mother care, so that every newborn can survive and thrive.

## Background

Severely sick newborns, including those with infections, severe intrapartum insults, severe jaundice or those who are too small to maintain their body temperature or to breathe or to feed actively, will require inpatient care to survive. This paper forms part of a series on high quality maternal and newborn care and examines bottlenecks and solutions specific to the provision of newborn inpatient care for small and sick babies.

The first 28 days of life is a vulnerable time for newborns, with an estimated 2.8 million babies dying during the first month of life worldwide in 2013 [1]. The main causes of death include direct complications of prematurity (36%), intrapartum events (previously called birth asphyxia) (23%), and infections (23%) [2,3]. Nearly three-quarters of all neonatal deaths occur in the first week of life [3]. The highest risk of death or serious morbidity occurs among the 10 million born at term with low birth weight (<2500 g) [4] and the 15 million born preterm (before 37 completed weeks of gestation) each year [5]. Many lives could be saved, and morbidity prevented, through a combined health systems approach [6] along the continuum of care, with identification of those at high risk and timely provision of quality inpatient and supportive care [7]. Strengthening of existing facility-based systems for the care of vulnerable newborns is the most effective approach for saving newborn lives [8] and is central to achieving the goals of the *Every Newborn *Action Plan (ENAP) [9].

**Figure 1:**
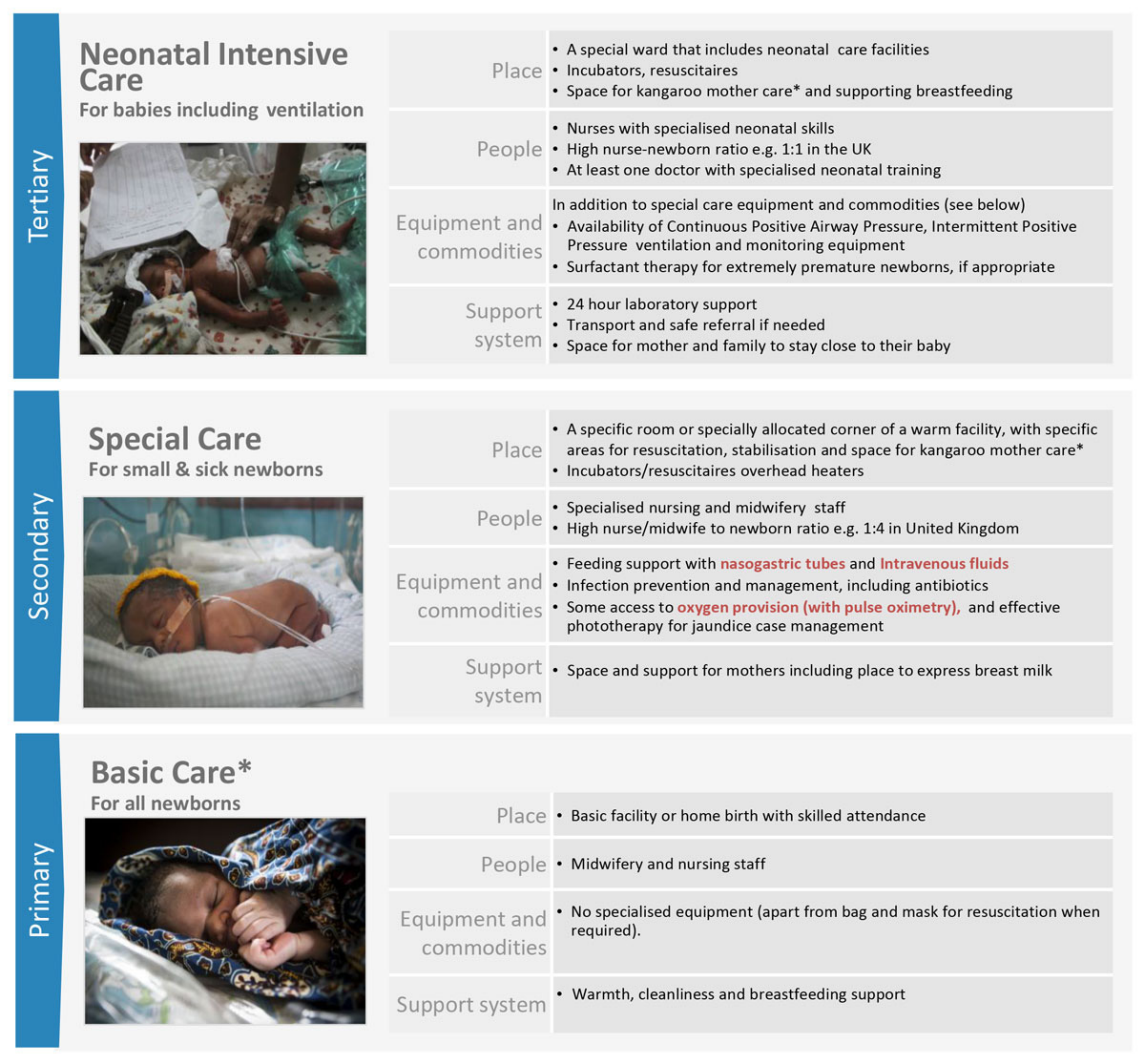
**Inpatient care of small and sick babies, showing health system requirements by level of care**. Red text signifies tracer indicator for bottleneck tool analysis. *See Vesel et al (2015) Kangaroo mother care, Enweronu-Laryea et al (2015) Basic newborn care and resuscitation, and Simen-Kapeu et al (2015) neonatal sepsis. Neonatal intensive care image source: Getty images/Save the Children. Special care for small and sick newborns image source: Ian Hurley/Save the Children. Basic care for all newborns image source: Jonathan Hyams/Save the Children.

**Figure 2:**
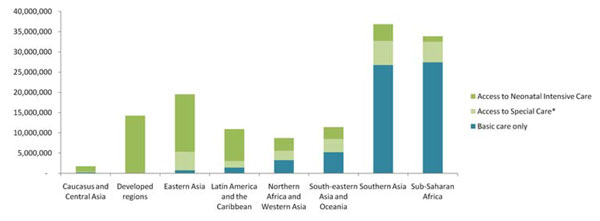
**Estimated coverage of neonatal care by region of the world and level of care**. *By Special Care Baby Unit, this is the highest level of care available (i.e. no Neonatal Intensive Care). Data source: Adapted from Beyond Newborn Survival: The Global Burden of Disease due to Neonatal Morbidity. Estimates of neonatal morbidities and disabilities at regional and global levels for 2010: introduction, methods overview, and relevant findings from the Global Burden of Disease study. Pediatric Research; December 2013, Volume 74, (Supplement 1).

Inpatient care is usually delivered across three levels (Figure 1) and refers to the facility-based care of newborns focused on both treatment and prevention of infection and further complications. Prevention includes protection from hypothermia (ensuring warmth) and hospital acquired infection, as well as the provision of adequate nutrition (often with nasogastric or cup feeding), with the overall goal of establishing exclusive breastfeeding where possible. Treatment, where available, centres on the management of common neonatal conditions including respiratory distress syndrome (RDS), neonatal infections, hyperbilirubinaemia, feeding difficulties [7] and the prevention and treatment of retinopathy of prematurity (ROP) [10]. Advanced treatment for other important conditions, such as necrotising enterocololitis (NEC), patent ductus arteriosis (PDA), correctable congenital anomalies and broncho-pulmonary dysplasia (BPD) may also be undertaken. Basic newborn care (providing cleanliness, warmth and support for breastfeeding) is essential for all babies, including timely resuscitation for up to 10% of babies that may require resuscitation at birth [11] and is covered elsewhere in this series [12]. Inpatient care for small or sick babies includes two cornerstone components: Kangaroo Mother Care (KMC) and sepsis case management, which are also considered elsewhere in this series [13,14]. While in a well-functioning health system all three levels of care will be available (Figure 1), many small babies can be managed without provision of any higher level neonatal intensive care and can be looked after in special care units [7]. Currently, however, over three quarters of babies born in Sub-Saharan Africa and Southern Asia cannot access special care if they were to require it (Figure 2).

High quality inpatient care for sick neonates includes careful monitoring by trained health professionals with a sound understanding of the physiological and psychosocial needs of the small or sick newborn baby and their families. A recent DELPHI exercise estimated that optimal supportive care in a hospital Special Care Baby Unit (SCBU) could avert 70% of neonatal deaths due to preterm birth complications, and that 90% could be averted with availability of hospital Neonatal Intensive Care Units (NICUs) [6]. Whilst coverage of these inpatient care packages are near universal in high-income settings, both the coverage and the quality of care available in middle-and low-income settings are highly variable [15]. The provision of high quality nursing and inpatient medical care of small and sick newborns not only saves lives, but could also help to facilitate more rapid discharges from health facilities, leading to better short and long-term morbidity outcomes for these babies, including reduction of BPD and ROP. This need is reflected by the current burden of long term disability in survivors following preterm birth being greatest in middle income countries, particularly where coverage of inpatient neonatal care has expanded without due attention to the quality of care provided [10].

Inadequate care in facilities can be caused by a number of constraints usually related to health worker shortages and poorly equipped facilities, compounded by a lack of specific knowledge and competencies in dealing with small and sick newborns amongst existing clinicians and nursing staff [9,16]. Facility-based neonatal care frequently remains under-prioritised and under-funded in many parts of the world, particularly in low and middle income countries (LMIC). Few standardised indicators exist to measure quality of newborn care in facilities and challenges remain to improve the metrics and core competencies [17]. Inadequacies in supplies and safe use of medicines and equipment (including effective phototherapy and case management for sick neonates) are common problems despite the fact that evidence-based interventions exist that can be delivered in resource-constrained environments [18].

The vision of providing quality care to sick newborns is part of a wider global movement - the United Nations (UN) Secretary General Global Strategy in 2010 [19] called for innovative approaches to provide quality care for mothers and newborns, using coordinated research and the formulation of accountability mechanisms through the Commission on Information and Accountability for Women's and Children's Health (COIA). Published in 2014, The Lancet Every Newborn Series (http://www.thelancet.com/series/everynewborn) demonstrated the progress that has been made, even in challenged settings, and outlined the urgent steps still needed to improve newborn survival. The Lancet papers proposed a package of integrated quality interventions [16,20] - the Every Mother, Every Newborn (EMEN) initiative - that have been outlined in the Every Newborn Action Plan (ENAP) alongside specific actions and ambitious targets for newborn survival [9]. This paper aims to interrogate country-level data on "bottlenecks" to quality care and to draw out innovative solutions, in order to aid the formulation of country led health plans and strengthen the capacity of health systems to respond to the needs of small and sick newborns.

Objectives of this paper are to:

1. Use a 12-country analysis to explore health system bottlenecks affecting the scale up of inpatient supportive care for small and sick newborns

2. Present the solutions to overcome the most significant bottlenecks including learning from the 12-country analyses, literature review and programme experience

3. To discuss policy and programmatic implications and propose priority actions for programme scale up.

## Methods

This study used quantitative and qualitative research methods to collect information, assess health system bottlenecks and identify solutions to scale up of maternal and newborn care interventions in 12 countries: Afghanistan, Cameroon, Democratic Republic of Congo (DRC), Kenya, Malawi, Nigeria, Uganda, Bangladesh, India, Nepal, Pakistan and Vietnam.

### Data collection

The maternal-newborn bottleneck analysis tool (additional file 1) was developed to assist countries in the identification of bottlenecks to the scale up and provision of nine maternal and newborn health interventions across the seven health system building blocks as described previously [16,20]. The tool was utilised during a series of national consultations supported by the global *Every Newborn *Steering Group between July 1^st ^and December 31^st, ^2013. The workshops for each country included participants from national ministries of health, UN agencies, the private sector, non-governmental organisations (NGOs), professional bodies, academia, bilateral agencies and other stakeholders. For each workshop, a facilitator oriented on the tool coordinated the process and guided groups to reach consensus on the specific bottlenecks for each health system building block. This paper, seventh in the series, focuses on the provision of inpatient care of small and sick newborns.

**Figure 3:**
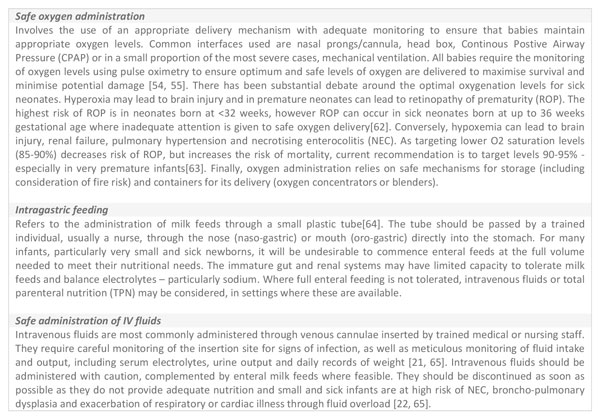
**Definitions of tracer indicators for inpatient care of small and sick newborn bottleneck analysis tool**. For more details see the complete bottleneck analysis in the additional file 2.

Tracer interventions were defined for each package to focus the workshop discussion. For the purpose of this bottleneck analysis, three interventions required for the treatment of common neonatal conditions were included as tracer items for the package of inpatient care: safe oxygen administration, intragastric tube feeding (IGTF) and the provision of intravenous (IV) fluids (Figure 3). Oxygen therapy is a mainstay treatment for small and sick babies, with respiratory compromise commonly seen in RDS (following preterm birth, neonatal pneumonia and neonatal sepsis) and respiratory failure being an important mechanism in most neonatal deaths [3]. Developmental immaturity of the preterm newborn (especially those born before 34 weeks gestation), or severe illness in a more mature neonate, may limit their ability to coordinate sucking and swallowing required for successful exclusive breastfeeding. In these instances, intragastric feeding is a commonly used low-tech intervention to deliver nutrition, using expressed breast milk where possible. In addition, many of the most small and sick newborns will require administration of IV fluids to prevent dehydration as a result of insensible water loss, and to manage the delicate fluid, electrolyte and glucose balance, especially in the first days after birth [21,22].

Safe implementation and monitoring of these interventions can be challenging, especially in low-resource settings. The list of tracers is not exhaustive and other important interventions, notably, effective phototherapy for the treatment of hyperbilirubinaemia (Figure S2, additional file 2), basic newborn care and resuscitation [12], KMC [13] and management of neonatal sepsis [14] are covered by other sections of the bottleneck analysis tool.

### Data analysis methods

**Figure 4:**
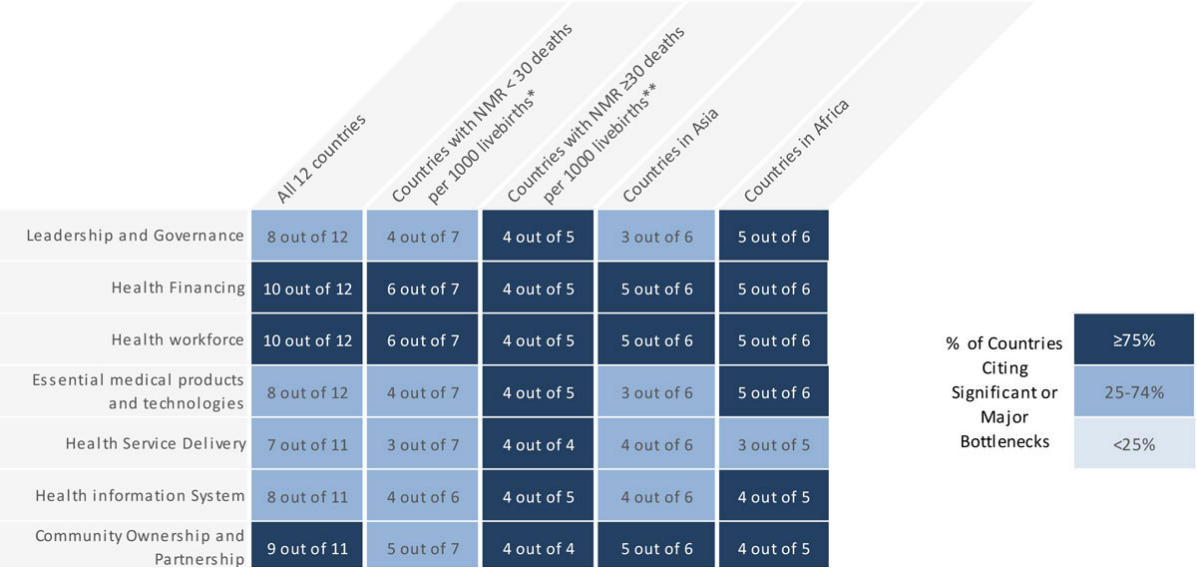
**Very major or significant health system bottlenecks for inpatient care of small and sick newborns**. NMR: Neonatal Mortality Rate. *Cameroon, Kenya, Malawi, Uganda, Bangladesh, Nepal, Vietnam. **Democratic Republic of Congo, Nigeria, Afghanistan, India, Pakistan. See additional file 2 for more details.

**Figure 5:**
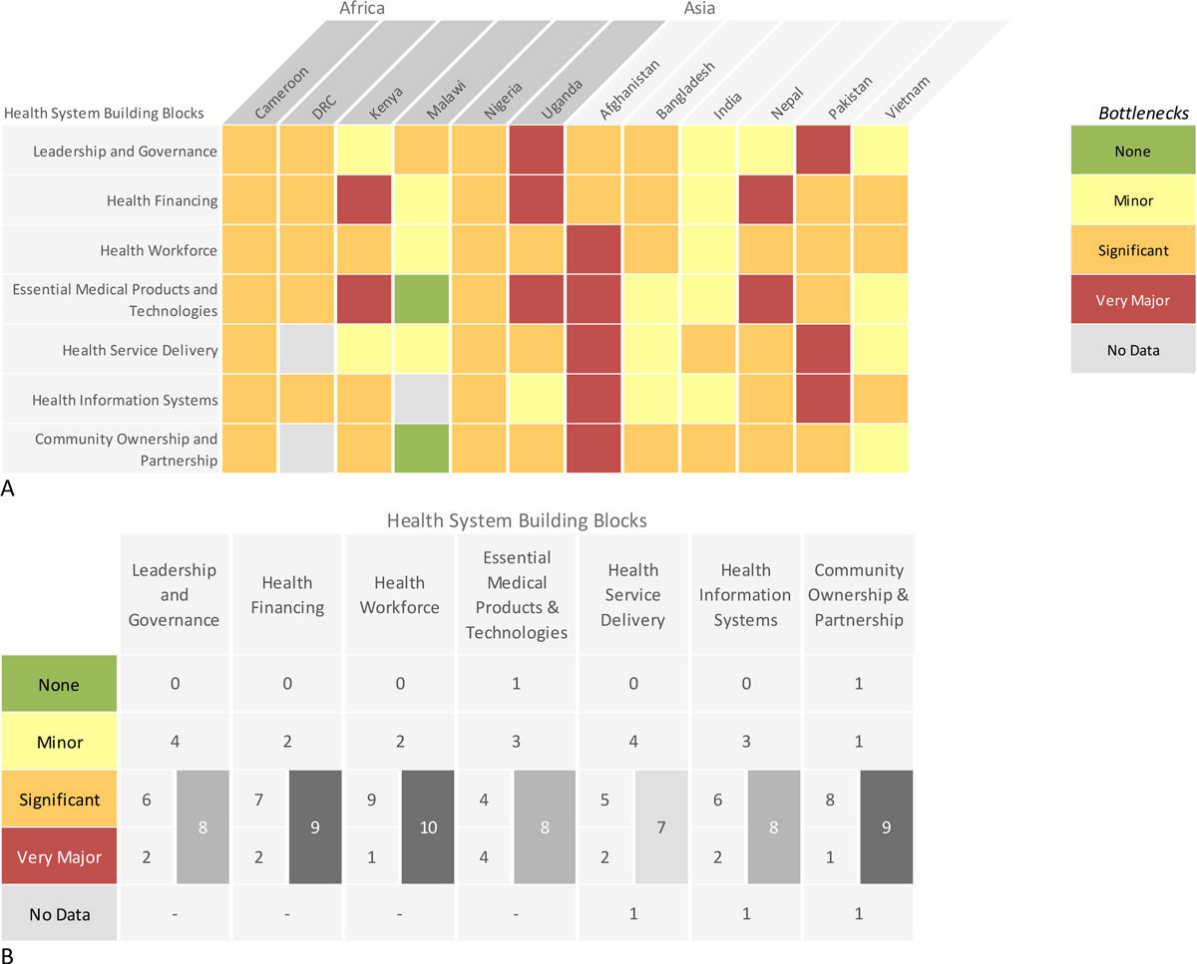
**Individual country grading of health system bottlenecks for inpatient care of small and sick newborns**. Part A: Heat map showing individual country grading of health system bottlenecks for inpatient care of small and sick newborns. Part B: Table showing total number of countries grading significant or major for calculating priority building blocks. DRC: Democratic Republic of the Congo.

Data received from each country were analysed and the graded health system building blocks were converted into heat maps (Figures 4 and 5). Bottlenecks for each health system building block were graded using one of the following options: not a bottleneck (=1), minor bottleneck (=2), significant bottleneck (=3), or **very **major bottleneck (=4) (Figure 5). We first present the number of countries from which workshops participants categorised health system bottlenecks as significant or very major, by mortality contexts (Neonatal Mortality Rate (NMR) <30 deaths per 1000 live births and NMR ≥30 deaths per 1000 live births) and region (countries in Africa and countries in Asia) (Figure 4). We then developed a second heat map showing the specific grading of health system bottlenecks for each country (Figure 5).

Context specific solutions to overcome challenges to scaling up inpatient care identified in all countries were categorised into thematic areas and then linked to the specific bottlenecks in the results section (Table 1/ Table S1, additional file 2). We undertook a literature review to identify further case studies and evidence-based solutions for each defined thematic area (Additional file 2). For more detailed analysis of the steps taken to analyse the intervention specific bottlenecks, please refer to the overview paper [20].

**Table 1:** Summary of solution themes and proposed actions for inpatient care for small and sick newborns.

Health system building blocks	Solution Themes	Proposed actions
Leadership and Governance	*Advocacy and political will* *Improve organisation structures* *Review and disseminate guidelines*	• Active involvement of national advocates (professional bodies, academic, policy makers) for care of sick newborns • Increase number of special care units and spaces in facilities for newborns • Develop national policies and guidelines for referral systems, organisational standards for sick newborn care

Health Financing	*Budget allocation* *Innovative funding and removal of user fees*	• Increase and sustain funding for sick newborns, earmark funds within facilities caring for newborns • Expand existing maternal health schemes (end-user incentives, insurance schemes, voucher schemes) to cover inpatient care of newborns • Long term vision and health systems approach towards universal coverage for healthcare

Health Workforce	*Recruitment and Retention* *Competency based training* *Task shifting*	• Develop neonatal nursing cadre with agreed standards and benchmarks • Strategies to incentivise neonatal health workers • Develop job descriptions, appropriate remuneration and career development pathways for health workers caring for newborns • Scale up of simplified, skilled based training programmes on infection prevention, feeding, provision of warmth and family centred care for newborns • Maximising existing resources, including nurses, lower level health workers and communities

Essential Medical Products and Technologies	*Essential medical list* *Logistic system strengthening and forecasting*	• Update and implement the essential medical list to include oxygen • Inclusion of neonatal equipment and drugs in logistics systems • Strengthen oxygen systems at national and local level

Health Service Delivery	*Increase service delivery and rationalise service distribution* *Quality improvement and assurance* *Improve working conditions*	• Special care baby units (or dedicated area) in every district hospital • Decentralisation of inpatient neonatal care, stable babies cared for in KMC units • Develop and harmonise quality assurance tools and carry out quality assessment of neonatal units • Provide supportive supervision and mentoring • Improve remuneration and incentives (see also, health workforce), working hours, food provision and facilities to stay

Health Information System	*Strengthening and integration of HMIS* *Development of indicator definitions, reporting systems, tools and* *Scale up audits and registers*	• Integrate newborn indicators into national health information systems • Define and harmonise newborn indicators, especially care of sick newborns • Regular mortality audits in all special care and neonatal intensive care units.

Community Ownership and Participation	*Accessibility of information and community awareness* *Improve care seeking and linkages* *Male involvement*	• Sensitisation on importance of newborn inpatient care and entitlements to care • Use of community volunteers, local champions and leaders • Develop local transportation solutions for families, improve patient experience in facilities and develop family-centred guidelines • Male role models in the community, inclusive policies and frameworks in facilities • Education on maternal and newborn health targeted at men

## Results

Our analysis identified bottlenecks across seven health system building blocks relating to the inpatient supportive care of small and sick newborns. Twelve countries submitted their responses to the inpatient care of small and sick newborns bottleneck tool. Afghanistan, Cameroon, Democratic Republic of Congo (DRC), Kenya, Malawi, Nigeria, Uganda, Bangladesh, Nepal and Vietnam returned national level responses. Pakistan provided subnational data from all provinces, Gilgit-Baltisan, Azad Jammu and Kashmir, excluding two tribal territories. India returned subnational data from three states: Andhra Pradesh, Odisha and Rajasthan.

DRC did not provide a grade for health service delivery and community ownership and partnership; and Malawi did not provide a grade for health information systems. In these cases the country was removed from the sample for the quantitative grading of that building block, but included for all other building blocks; their examples of described bottlenecks were still included in the analysis and presented in the results. Afghanistan listed their bottlenecks and completed rating for all building blocks, but did not propose any solutions.

The solution themes are summarised by health system building block in Table 1 (with more details in additional file 2). Care of small and sick newborns is a newborn intervention area highlighted by all country workshop participants as a major challenge to health systems, especially when considered in comparison with other intervention areas studied in the workshop. Grading according to the number of countries that reported very major or significant health system bottlenecks for inpatient supportive care for small and sick newborns is shown in Figure 4. Overall, the health systems building blocks with the most frequently reported very major or significant bottlenecks were health financing (10 out of 12 countries), health workforce (10 out of 12 countries), followed by community participation (9 out of 11 countries), suggesting these may be priority areas within which to tackle barriers to the scale up of inpatient care for small and sick newborns. As expected, building blocks were rated more poorly in countries with higher NMR. African countries reported a higher number of major and significant bottlenecks, but Afghanistan had the highest level of very major bottlenecks and Malawi had the lowest graded bottlenecks, as shown in Figure 5.

## Leadership and governance bottlenecks and solutions

The first building block, leadership and governance, was considered to have very major or significant bottlenecks across 5 of the African countries, and 3 of the Asian countries (Figure 4). Countries in both regions commonly identified a lack of national level advocates (including policy makers, key individuals within professional bodies, academics and national institutions) for advancement of quality care for newborns. At the governance level, country workshop participants highlighted lack of supportive policies for care of small and sick babies. Specifically, workshop participants noted that their existing policies were not inclusive of the key supportive and organisational policies for newborn care, such as well-defined, rational referral systems, discharge criteria and standardised levels of care at the district and peripheral level. Policy documents in circulation amongst senior officials were not always disseminated to the managers at lower levels of the health service and did not always incorporate guidelines with important components of special care for newborns, such as supportive policies, guidelines for breastfeeding and family centred care (Table 1/ Table S1, additional file 2).

Solutions proposed by country teams centred on the need for targeted advocacy and political will. They focused on improving the organisational and supportive structures for sick newborns at the policy and governance level and building local champions. Country workshop teams proposed reviewing the existing organisational policies and guidelines at a central level and ensuring these were disseminated to all levels of the health system (Table 1/ Table S2, additional file 2).

## Health financing bottlenecks and solutions

Health Financing bottlenecks were frequently graded as needing significant work for inpatient care of newborns - 10 out of all 12 country teams (Figure 4) graded it as very major or significant, with only Malawi and India perceiving there to be only minor bottlenecks (Figure 5). Revenue collection for newborn health, and competing calls for financing of other areas of healthcare, was clearly viewed as a barrier, and insufficient earmarked funds at the facility was impeding their ability to provide quality care to sick newborns. Participants specifically described a lack of designated funding for laboratory support and to purchase supplies such as blood components, antibiotics and other equipment for newborns, such as oxygen cylinders. The most frequently described health financing challenges pertain to prohibitive user-fees and insurance policies that do not cover inpatient care of newborns showing that families are frequently put at risk of severe financial hardship in the event of a baby being born small or sick (Table 1/ Table S1, additional file 2).

Country workshop participants proposed solutions including the need to increase amount of earmarked funding available for sick newborns and the need to mobilise and advocate for increased funding at the health system level. Participants also proposed more innovative funding mechanisms in order to remove the prohibitive user fees placed on care of sick newborns, either through more comprehensive health insurance, community-based finance or mutual health schemes (Table 1/ Table S2, additional file 2).

## Health workforce bottlenecks and solutions

Almost all countries identified the lack of trained personnel in neonatal care in quantity and quality (knowledge, training, skills) and 10 out of 12 graded these bottlenecks as significant (Figure 4), with Afghanistan grading their bottlenecks as very major (Figure 5). Poor supervision and the need for specialist and refresher training in neonatal skills were overarching challenges. Countries described difficulties recruiting specialist staff to work in remote areas and staffing disparities between urban and rural areas; 8 countries specified that problems in the health workforce stemmed from the lack of competency-based training and refresher training for the health workforce managing small babies, especially at the lower levels of the health system. Regarding task shifting, some countries noted that often only physicians are authorised to carry out tasks that could be performed by lower level health workers, such as prescribing oxygen or antibiotics. Other countries indicated that job descriptions were not clear in roles and responsibilities for those providing care to sick newborns, which is particularly relevant for neonatal nurses. Country workshop participants underlined that the motivation for neonatal nurses and other professionals to provide high quality care to sick babies was low (Table 1/ Table S1, additional file 2) and that incentives and remuneration were insufficient, leading to poor health worker attitudes, ineffective communication and poor compliance with infection control procedures.

Participants recognised that to remove health workforce bottlenecks, detailed health worker mapping of those caring for sick newborns was needed to identify the resources available and where tasks could be rapidly shifted to make more rational use of the existing workforce. Workshop participants also proposed improving working conditions, motivation and skills through more structured pre-service and in-service training and more appropriate remuneration for neonatal skills, including rewarding those prepared to work in rural areas (Table 1/ Table S2, additional file 2).

## Essential medical products and technologies bottlenecks and solutions

The provision of essential medical projects and technologies was graded as having very major bottlenecks by a third of all country workshop participants (Figure 4). The Essential Medicine List (EML) was a commonly described bottleneck; participants noted that the EML lacked the commodities required for special care of newborns, such as oxygen and IV fluids and was not implemented at the national level. Many participants described general stock-outs of neonatal equipment, especially cannulas and drugs (specifically antibiotics) and lack of availability of specialist equipment, such as continuous positive airway pressure (CPAP) and portable radiographs. Participants reported that weak and inaccurate information systems underpinned this problem, limiting the ability of facilities to forecast the demand for oxygen, fluids and the maintenance supplies needed for provision of quality inpatient supportive care (Table 1/ Table S1, additional file 2).

Solutions to the essential medical products and technology bottlenecks started with a need to update the EML to reflect the essential commodities needed for sick newborns (oxygen, antibiotic and IV fluids). Following this, workshop participants recognised a need for improving and building logistics management capacity to support the health system to manage inventories and prevent stock-outs (Table 1/ Table S2, additional file 2).

## Health service delivery bottlenecks and solutions

Service delivery was described as a challenge in all the countries with higher mortality contexts (Figure 4). Workshop participants described the limited number of facilities available to provide any type of services or inpatient care for sick or low birth weight babies, particularly at lower levels of the system. Poor enabling environments, undersized and outdated buildings, and lack of resource capacity for both delivery of care and provision of family-centred supportive care for babies in the public sector were commonly described. Five countries highlighted the limited space in health facilities for the special care of sick newborns. This included potential space for mothers to stay with their baby or lack of nurseries or side rooms for sick babies. Other country workshop teams described quality improvement as a major challenge due to inadequate monitoring or lack of quality improvement tools, poor mentoring and supervision, and poor implementation of clinical guidance and cot-side care plans for all staff caring for newborns (Table 1/ Table S1, additional file 2).

Country workshop participants recognised that the number of facilities or, at least, dedicated spaces for sick newborns needed to be increased and that service delivery needed to be rationalised. In alignment with the health workforce bottlenecks, teams suggested that quality assurance tools, quality improvement strategies (including care protocols), and improved mentorship and supervision for those delivering care to newborns could help to improve service delivery (Table 1/ Table S2, additional file 2).

## Health information system bottlenecks and solutions

The lack of health information and standardised, well-defined indicators to measure interventions for sick newborns is a central issue being tackled within the ENAP [9]. Most participants from higher mortality contexts graded it as a significant or very major bottleneck to the provision of quality care in facilities (Figure 5). Specific barriers to quality improvement in facilities included the absence of effective mortality audits in facilities, lack of both coverage and process indicators and registers on sick newborns with the existing data were not well managed. In other settings, participants recognised the need for strengthening and integration of newborn facility-based care indicators into their national HMIS (Table 1/ Table S1, additional file 2).

Country workshop participants stated a need for clear definitions for indicators and harmonising these indicators such that national Health Management Information Systems (HMIS) can be strengthened and include select indicators for sick newborns. This would require improved measurement tools, reporting systems and use of appropriate software. Participants highlighted a need for capacity building within health information to support the appropriate disaggregation, dissemination and reporting of sick newborn data. Teams also suggested scaling up regular mortality audits for neonatal units (Table 1/ Table S2 and S3, additional file 2).

## Community ownership and partnership bottlenecks and solutions

The community ownership and partnership building block was graded as having significant or very major bottlenecks in three-quarters of countries (Figure 4). Malawi was the only country for which workshop participants graded this building block as having no bottlenecks (Figure 5). Workshop participants specified a wide ranges of issues largely related to a lack of general information and awareness in communities about sick babies. Limited knowledge of the treatment processes and the severity of newborn illness, including poor awareness of the civil rights of babies born sick or low birth weight to access care, were highlighted. There were a number of access related problems reported, including poor referral and transport systems and inability to access facilities either due to cost or availability. For mothers in the community, participants described the lack of female decision-making power, loss of wages due to caring for a sick newborn and lack of privacy in facilities (Table 1). Lack of involvement of men was mentioned by six countries partially related to poor awareness and engagement of the wider community on issues related to sick newborns (Table 1/ Table S3, additional file 2).

Solutions for community ownership were wide-ranging, but were themed around improving the accessibility of information for carers and the services for small and sick newborns. Participants suggested a need for greater community awareness of the needs for sick and small newborns in order to improve demand, compliance and patient experience; specifically, encouraging male involvement and increased participation of the community in processes to improve family centred care in facilities (through development of materials, tapping into community groups and developing mutual health type schemes) (Table 1/ Table S2, additional file 2).

## Discussion

This paper has presented an analysis and synthesis of bottlenecks and solutions for one of six key intervention packages to reduce neonatal mortality worldwide reviewed in this series of papers; inpatient care for small and sick newborns. Previous analysis of the bottleneck data showed that amongst all intervention packages explored, inpatient care has some of the highest graded bottlenecks hindering scale-up [16], with very major or significant bottlenecks being reported across all health systems building blocks. Whilst inpatient care for the small and sick newborn forms part of the overall care along the continuum from pre-pregnancy to childhood, these findings are timely and this issue is new on the global agenda. Complications from preterm birth are now the leading cause of death in children under five [1]. Previous experience from high income settings has shown that initial provision of low-tech supportive inpatient care and case management, followed by full high-tech neonatal intensive care, has played an important role in reducing overall neonatal mortality [23]; therefore, in order to further reduce the burden of death due to prematurity, strategies to provide comprehensive, high quality inpatient care for small and sick newborns must be developed.

The methodology used in the bottleneck analyses employed a unique consultative and participatory approach to bring together a wide range of partners and players in newborn health. Rather than the top down approach employed by many research initiatives, this data collection and analysis methodology focused on eliciting information from ground-level field implementation, as perceived by stakeholders and experts in 12 countries with the highest burden of neonatal mortality. This has helped the data to capture context specific challenges and has enabled participants to share their experiences and work together to identify innovative solutions. The grading process encouraged the workshop participants to reach consensus on the perceived challenges and generate a quantitative measure of the perceived bottlenecks to delivering care to this vulnerable sub-population. Rather than reporting on systematic reviews or results from randomised trials, this paper aims to facilitate programmatic learning through the South-to-South exchange. This paper has brought together a wide range of programmatic experience and technical expertise in neonatal care from across the globe to inform programme managers and policy makers in multiple settings facing a range of health system challenges in delivering high quality, facility-based care to small and sick newborns.

Health systems seek to ensure that individuals in need of care receive high quality health services without the risk of financial catastrophe. This analysis identified three priority health systems building blocks with substantial barriers to implementation of facility-based care for small and sick newborns: health workforce and health financing followed by community ownership and partnership. Solution themes, including examples from literature review and programme learning, are discussed in detail below.

### Health workforce priority actions

**Figure 6:**
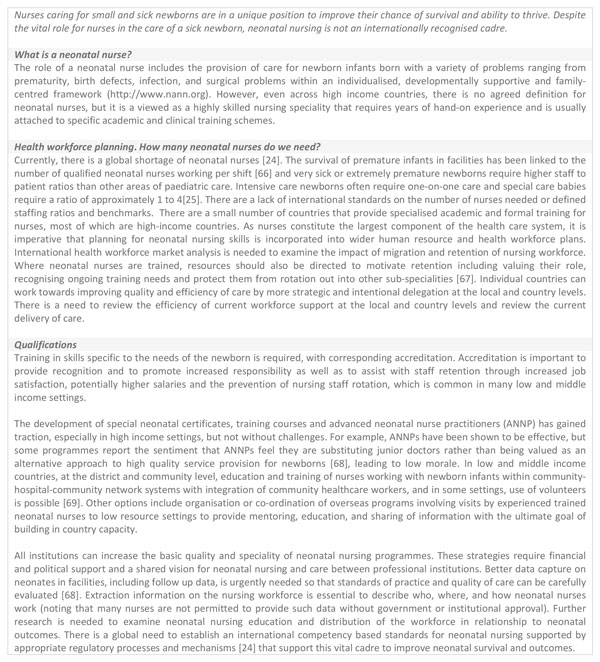
**Neonatal nursing as part of national human resource planning**. ANNP: Advanced Neonatal Nurse Practitioner.

A worldwide nursing shortage exists in both high and low resource settings [24,25]. For small and sick newborns this is not simply a shortage of qualified individuals; there is a critical human resource gap for a neonatal nursing cadre, with almost no neonatal nursing training programmes outside of high income countries (Figure 6). Neonatal nurses are the backbone of newborn inpatient care, as both providers of frontline care to the newborns and their families, but also through extended roles such as the advanced neonatal nurse practitioners (ANNPs) [26,27]. To improve neonatal outcomes, particularly in those countries which account for the highest newborn death and morbidity rates, nurses need to be recruited and offered specialised training in how to care for small and sick newborns, and be provided with ongoing resources to enable them to give consistent high quality care. There are other factors at institutional and country level including inadequate allocation of resources for a health workforce, inadequate workforce planning, poor retention strategies, ineffective use of existing nursing staff, and poor working conditions [16,28].

#### Skills-based/competency based training

Almost all countries in the workshop highlighted the lack of skills-based training programmes for health workers caring for small and sick babies. Qualitative work on the barriers to nurse education for those caring for sick newborns has found that educational programmes focusing on neonatal skills are often inconsistent, poorly structured, or may require long, off-site training courses making them inaccessible for large numbers of lower level hospitals or SCBUs [29]. Survive and Thrive is a private and public partnership with the American Academy of Pediatrics and has developed educational programmes focused on newborns. Essential Care of the Small Baby (ECSB) [30] is to be released in early 2015 and addresses skills such as nasogastric feeding and prevention of infection and skin-to-skin care through a cooperative learning approach. Learning techniques used by ECSB are skills-based and focused on small group work, using simulation methodology and role-play to practice technical and communication skills. Knowledge is tested through multiple-choice questions and Observed Structured Clinical Evaluations (OSCEs). Such pre-service and in-service training programmes are available and could be scaled-up within health worker training, even in lower resourced settings, as they do not rely on electricity supplies (being flip-chart based) and make use of low-cost simulation models. Well-designed programmes focused on neonatal clinical skills have been shown to be effective and improve health provider knowledge and practice [31], but will require supervision systems and regular refresher training to sustain and update skills [32].

#### Task shifting

The World Health Organization (WHO)'s recommendations on optimising the roles of health workers aim to address critical health workforce shortages that slow progress towards the health-related Millennium Development Goals [33]. A more rational distribution of tasks and responsibilities among cadres of health workers can significantly improve both access and cost-effectiveness - for example, by training and enabling 'mid-level' and 'lay' health workers to perform specific interventions otherwise provided only by cadres with longer (and sometimes more specialised) training. These recommendations are intended for health policy-makers, managers and other stakeholders at a regional, national and international level. WHO hopes that countries will adapt and implement them to meet local needs. The recommendations were developed through a formal, structured process including a thorough review of available evidence. Specific examples that have been taken up include nursing auxiliaries or health care assistants supporting and maintaining KMC [33]. In Malawi, ward attendants have been involved in supporting KMC [34] and health surveillance assistants have been trained to promote facility-based care for sick newborns [35]. ECSB training incorporates task shifting to mothers, when appropriate, for basic skills such as nasogastric feeding and providing basic care to a small baby looked after in a facility [30].

#### Recruitment and retention

Once health workers have the skills needed to care for small and sick babies, recruitment and retention strategies are needed to supervise and motivate, which is especially important for rural and hard to reach postings. Innovative recruitment and retention strategies have been implemented with success in some settings. Thailand has historically used a bonding system to improve recruitment of health workers for rural areas. Newly qualified health professionals, including doctors and cadres of nurses are required to spend a mandatory time period in rural postings. On completion, professional qualifications can be upgraded. Evidence suggests this has led to a substantial increase in the numbers of trained professionals in rural areas and is partially responsible for the impressive health gains in Thailand in the last 25 years [36,37].

In addition to task shifting there are other immediate, interim strategies that can be put in place. These could include improving conditions for the workforce through incentives [38] (financial, educational or other), relieving staff of other duties, improving daily working conditions (break areas, food vouchers, accommodation on-site or nearby) [39] and improving job satisfaction through structured supervision and mentoring efforts [32]. Non-rotation of staff out of neonatal care is an important strategy to prevent neonatal staff being shifted annually within the hospital from department to department or into other specialties (Figure 6).

### Health financing priority actions

#### Budget allocation

Whilst the health financing issues faced by many low-income countries are due to the lack of financial resources for health and development overall, and are not unique to the newborn [40], those newborns requiring inpatient care are at greater risk due to their need for specialised facility-based care. Newborns are relatively neglected in official development assistance [41] and specialised, intensive care is often perceived as prohibitively expensive. A strong economic case, including the relative burden of newborn mortality globally, and the argument for prevention of long-term morbidities, is required to advocate for the earmarking of funds specifically for developing and sustaining high quality inpatient newborn care. The issue of health financing is explored in greater detail in paper 1 of this series [20].

#### Innovative funding and removal of user fees

The birth of a small or a sick baby can be financially catastrophic for families. Shifting from a reliance on out-of-pocket payment to prepayment and risk pooling is a critical part of the health financing transition that most countries go through as they get richer [42]. Limited risk pooling means that insurance and depth of coverage is a common problem for families. Removal of user fees in the public sector is a first step, but has associated risks and challenges and must be replaced by alternative health financing mechanisms that could include: social health insurance, community based health insurance and government supply side financing [43]. The success of these schemes is dependent on the context within the countries where they are implemented. Rwanda's community financing scheme is backed by compulsory government payments into the scheme and stringent pooling of donor funds [44]. Provision of coverage for inpatient newborn care within insurance schemes or voucher and incentive systems is a neglected area, with often only delivery and basic newborn care being covered. Attention to successful schemes that already exist in countries could partially ameliorate the risk of financing catastrophe for families when a baby is born small or sick, rather than introducing new schemes for sick newborns that may further fragment health financing systems. Sick newborn care is frequently not covered by maternity packages or maternal health financing schemes (e.g. Nepal vouchers scheme), yet has potentially large expenses associated with it. Schemes using prospective case-based systems for inpatient care - as in Kyrgyzstan [36] could be adapted to give higher priority to newborn inpatient and special care. Further implementation research is needed for innovative funding mechanisms to identify factors that may facilitate their success and provide recommendations for their implementation in different settings.

### Community ownership and partnership priority actions

Whilst reported bottlenecks to high quality inpatient newborn care are similar across regions, individual communities differ in their geographical and socio-cultural structures and available resources. Enabling maximum effect through tailor-made solutions for a given community will require empowering solutions from a grassroots level.

#### Community awareness

Lack of demand for quality newborn inpatient care may reflect the fatalistic assumption that all small and sick babies will die [27]. Across settings, country teams highlighted the lack of awareness in communities about sick newborns, the treatment processes and their civil rights to access health services. Most country teams reported a lack of awareness of the severity of newborn illness and knowledge that timely, high quality care can save newborn lives. In some contexts, such as India, there are specific care-seeking barriers for newborn girls. The workshops participants' perceptions strongly suggest there is a lack of strategic, targeted health education on newborn health across settings and that sensitisation and local community education efforts are needed to reduce fatalism and increase care-seeking and demand. Mobilisation of communities using women's community groups has been shown to have a positive effect on a range of maternal and newborn health outcomes, including the potential to reduce neonatal mortality in a number of settings [45-47]. There is a clear role for community volunteers, local role models and community leaders to raise awareness on issues surrounding newborn health and the care of sick newborns.

#### Improve care seeking and transport linkages

Qualitative study of the local barriers and solutions for care-seeking in child health in Kenya, Nigeria and Niger highlighted important factors on perceived awareness and the subsequent demand for care [48]. Lack of trust in health services, perceptions that treatment is ineffective and experience of poor quality of care were perceived as important in reducing demand for care. Health services that are out-of-stock, negative experiences with health workers, or poor communication between staff and families, especially mothers, may be detrimental to the care of the newborn. Facilities may need to focus on community strategies to improve the patient experience in facilities, especially for mothers. It is critical for the mother to spend time with the sick newborn wherever possible, therefore, local hospital policy guidelines that encourage family-centred care and take into account the local and cultural family structure are vital for mothers to be able to participate in the care of their newborns. Local transport systems are needed to facilitate access between the community and facility, especially when newborns are in the facility for long periods of time. Within the facility, task shifting to mothers, in addition to the necessary support for breastfeeding and expressing milk, can play an important part in empowering mothers and securing the linkages between the family and inpatient care [49,50].

#### Male involvement

Half of the countries in the workshop specifically reported that there was a lack of male involvement in the care of sick newborns. Individual, family, community, societal and policy factors are previously identified barriers to male involvement during pregnancy and birth [51]. Qualitative research suggests men often lament their lack of involvement or understanding of maternal and newborn health issues [48] - an area that is often seen as dominated exclusively by females. Empirical research confirms that for pregnancies that are wanted and where men are more educated, men are more likely to be involved in maternity related care [52]. The care of sick newborns is no different and tackling barriers to male involvement is an issue that spans the care continuum from family planning to the care of a sick newborn in a facility. Men often control family finances or have a stronger influence on decision-making. Women may be removed from their usual schedules when their newborn is sick, leading to potential for neglecting other commitments (whether work or household related) and, therefore, may need additional support. Use of male role models in the community may help to facilitate this transition away from maternal and newborn health being viewed as an exclusively female domain. Using lessons learned from Prevention of Mother To Child Transmission (PMTCT) research [53], interventions to increase male involvement in newborn care include addressing hospital policies and staff attitudes in facilities to allow for culturally sensitive, inclusive policies for men and families, such as special visiting hours and supporting fathers to participate in KMC [13].

### Other priority actions

As highlighted in the analysis, very major or significant bottlenecks were reported across all building blocks. Solution themes for three of these building blocks have been discussed in detail above and more details on the country-specific bottlenecks for each health system building block are available in the additional file 2. A few other bottlenecks described were especially relevant to inpatient care. For example, India and Pakistan stressed the shortfall in supply of oxygen due to demand and supply gaps. Improving oxygen systems within health facilities is key to enable widespread availability when required. Oxygen cylinders are still commonly used in many facilities in low and middle-income settings, however they are expensive, require filling up regularly and are difficult to transport. Where power supplies are reliable, oxygen concentrators can provide a consistent and inexpensive source of oxygen. In view of the emerging epidemic of ROP [10], the use of oxygen in any setting should be carefully monitored using pulse oximetry and safe delivery mechanisms to ensure optimum and safe saturation levels [54,55], as described in Figure 3. The safe and systematic use of oxygen, as with all drugs, needs to involve training and supervision of nurses, doctors, technicians and administrators [56] and appropriate documentation is needed. Commonly prescribed antibiotics for small and sick newborns, such as gentamicin, which has potentially adverse effects related to dosage and interval [57] need particular attention to safety, especially where therapeutic drug monitoring is not possible [58]. A number of country teams highlighted newborn inpatient care health information bottlenecks. A recent assessment of facility-based neonatal care in Kenya highlighted how poor data were potentially undermining the quality of practice [59], especially affecting the assessment of gestational age and symptoms of severe illness. At a national level, efforts are needed to strengthen the HMIS and to develop basic indicator definitions for monitoring inpatient care with core competencies and standards for small and sick newborns by levels of care [17]. At the facility level, there is a clear need for improved documentation, registration and incorporating the use of regular mortality audits [60].

### Limitations

The data generated from the workshop came from the subjective and consensus views of participating national stakeholders, including government representatives and experts. The quality and amount of information extracted from these workshops varied depending on the level of knowledge of participants about health system issues and facilitation. In addition, bottlenecks were reported as perceived bottlenecks relative to the other health system building blocks under exploration. There may be instances where known health system challenges or deficits based on robust quantitative data may be in conflict with the perceived bottleneck grading. This may be due to the method of grading relative to other health system building blocks, or that participants place higher subjective value on other areas of their health system. An additional explanation is that groups' may view certain building block areas as easier challenges to overcome based on their knowledge of their setting and expertise in the specific newborn intervention being discussed. The tool is comprehensive and detailed, which is one of its strengths. However, it also may have caused some *workshop fatigue*, particularly towards the end of the workshop where teams discussed and recorded solutions. For example, for the inpatient care questionnaires, Afghanistan completed the bottleneck portion of the questionnaires, but did not submit any solutions. The analysis focused only on three tracer items: safe oxygen, IGTF and the provision of IV fluids. Other specific components of inpatient neonatal care may have different bottlenecks and solutions, for example, identification of and effective phototherapy for neonatal hyperbilirubinaemia [61] (Figure S2, additional file 2).

### Future agenda

**Figure 7:**
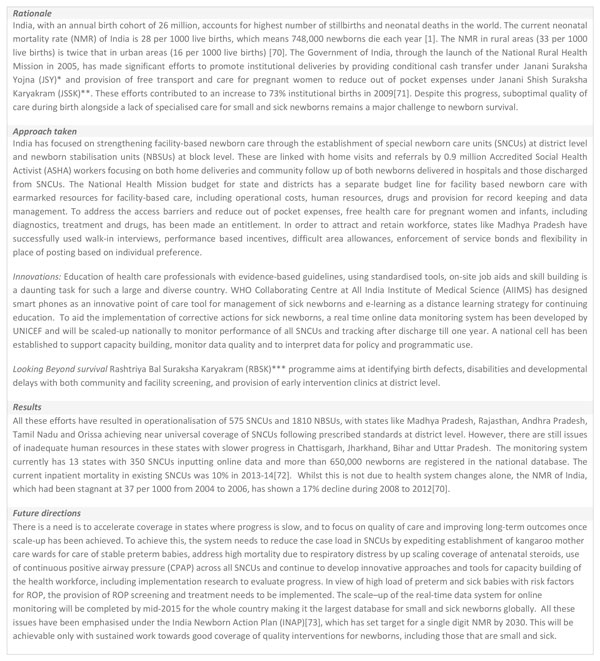
**India's health systems approach to improving inpatient care for small and sick newborns**. *Janani Suraksha Yojna (JSY): a conditional cash transfer to promote institutional delivery); **Janani Shishu Suraksha Karyakram (JSSK): reducing out of pocket expenses by making free health care an entitlement; ***Rashtriya Bal Suraksha Karyakram (RBSK): looks at developmental delays and disabilities, birth defects and deficiencies, covering age group of 0-18 years of age. Other abbreviations: AIIMS: All India Institute of Medical Science; ASHA: Accredited Social Health Activist; CPAP: Continuous Positive Airway Pressure; India Newborn Action Plan (INAP); NMR: Neonatal Mortality Rate; NBSU: Newborn Stabilisation Units; ROP: Retinopathy of Prematurity; SNCU: Special Newborn Care Unit; UNICEF: United Nations International Children's Emergency Fund; WHO: World Health Organization.

Improving inpatient newborn care will require a health systems approach and some countries are recognising this need. For example, the securing of political, professional and financial commitment in India has led to substantial increases in provision of quality inpatient newborn care (Figure 7). Previously, particularly in low-income settings, much investment has occurred in delivering public health and community-based interventions to improve newborn outcomes. This has led to important gains in outcomes, especially in settings with the highest neonatal mortality rates. However, as seen historically in high income countries, to reduce neonatal mortality further, attention is first required on improved supportive case management (which for the smallest and sickest newborns will require inpatient care) and then should be followed by the introduction and scale-up of neonatal intensive care [62].

**Figure 8:**
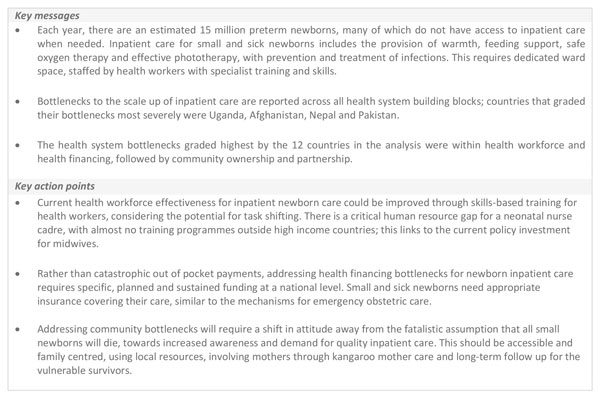
**Key messages and action points for inpatient care of small and sick newborns**.

Specific areas for action have been highlighted above, with many of these bottlenecks being critical to address to enable provision of quality inpatient newborn care (Figure 8). Interdisciplinary linkages and a focus on better quality data will help identify areas for improvement so that teams delivering care to small and sick newborns can plan and implement changes. Ongoing data monitoring helps the team recognise their improvement and identify specific areas to focus on in the future, so that the exercise is an ongoing cycle. The EMEN package [16] will be crucial to this process.

## Conclusions

Whilst major bottlenecks to the scale-up of quality inpatient newborn care are present, in many cases, effective solutions exist. Currently, there is a large grass roots commitment to improving care around the time of birth to end preventable maternal and newborn deaths and stillbirths, and to improve healthy outcomes as part of the ENAP [9]. Improving availability and quality of inpatient newborn care has been identified as an important area to achieve the aims of this plan, providing potential for political, professional and financial support to develop and scale-up solutions to these bottlenecks. We must build on this momentum, using knowledge of what works to ensure action, so that every small and sick newborn baby has access to timely, high quality and family-centred inpatient care as required to survive and thrive.

## List of abbreviations

ANNP: Advanced Neonatal Nurse Practitioner; BPD: Broncho-Pulmonary Dysplasia; COIA: Commission on Information and Accountability; CPAP: Continuous Positive Airway Pressure; DRC: Democratic Republic of Congo; ECSB: Essential Care of the Sick Baby; EMEN: Every Mother, Every Newborn; EML: Essential Medicines List; ENAP: Every Newborn Action Plan; HIC: High income countries; IGTF: Intra Gastric Tube Feeding; IV: Intra-Venous; JSSK: Janani Shishu Suraksha Karyakram; KMC: Kangaroo Mother Care; LBW: Low birth weight; LIC: Low income countries; LMIC: Low and Middle Income Countries; NBSU: Newborn Stabilization Unit; NEC: Necrotising Enterocolitis; NICU: Neonatal Intensive Care Unit; NMR: Neonatal Mortality Rate; NGO: Non-Governmental Organisation; OSCE: Observed Structured Clinical Evaluation; PDA: Patent Ductus Arteriosus; PMTCT: Prevention of Mother to Child Transmission (PMTCT); ROP: Retinopathy of Prematurity; RDS: Respiratory Distress Syndrome; SCBU: Special Care Baby Unit; SNCU: Special Newborn Care Unit; UN: United Nations; UNICEF: United Nations International Children Emergency Fund; WHO: World Health Organization.

## Competing interests

The authors have not declared competing interests. The assessment of bottlenecks expressed during consultations reflects the perception of the technical experts and may not be national policy. The authors alone are responsible for the views expressed in this article and they do not necessarily represent the decisions, policy or views of the organisations listed, including WHO.

## Authors' contributions

SGM was responsible for the analysis and writing process with HB and JEL who oversaw the analysis, writing and reviews of the paper drafts. KED along with the ENAP & UNICEF teams, were responsible for the overall coordination of the bottleneck analysis tool development, country consultation process, and reviews of the paper drafts. AS-K was responsible for the tool development and substantial contributions to the data analysis. All named authors contributed sections of text and approved the final manuscript.

## Supplementary Material

Additional file 1 Bottleneck tool questionnaire. Click here for file

Additional file 2 Supplementary tables, figures and literature search strategy. Click here for file

## Declarations

Publication costs for this supplement were funded by the Bill and Melinda Gates Foundation through a grant to US Fund for UNICEF (Grant ID: OPP1094117), and support from Save the Children's Saving Newborn Lives Programme. Additional funding for the bottleneck analysis was received from USAID (Grant ID: GHA-G-00-07-00007) through UNICEF.

This article has been published as part of *BMC Pregnancy and Childbirth *Volume 15 Supplement 2, 2015: Every Woman, Every Newborn. The full contents of the supplement are available online at http://www.biomedcentral.com/bmcpregnancychildbirth/supplements/15/S2.
