# Supplementary material for: Inpatient care of small and sick newborns: a multi-country analysis of health system bottlenecks and potential solutions
Source: BMC Pregnancy Childbirth. 2015 Sep 11;15(Suppl 2):S7. doi: 10.1186/1471-2393-15-S2-S7 (PMC4577807; doi:10.1186/1471-2393-15-S2-S7)
Supplement: Additional file 2 — Supplementary tables, figures and literature search strategy. [file 1471-2393-15-S2-S7-S2.docx]

Inpatient care for small and sick newborns: a multi-country analysis of health system bottlenecks and solutions

Additional file 2

A. Table S1: Bottlenecks for inpatient care of small and sick newborns 2

B. Table S2: Solutions for inpatient care for small and sick newborns 16

C. Figure S1: Subnational grading of bottlenecks for inpatient care of small and sick newborns 28

D. Literature Search Strategy 29

E. Figure S2: Overcoming bottlenecks for the management of neonatal jaundice: diagnostics and devices 31

F. Table S3: Significant bottlenecks and solution themes by health systems building block 32

G. References 38

# A. Table S1: Bottlenecks for inpatient care of small and sick newborns

| Healthy System Building Block | Category | Bottlenecks | Africa | | | | | | Asia | | | | | |
| --- | --- | --- | --- | --- | --- | --- | --- | --- | --- | --- | --- | --- | --- | --- |
|  |  |  | Cameroon | DRC | Kenya | Malawi | Nigeria | Uganda | Afghanistan | Bangladesh | India | Nepal | Pakistan | Vietnam |
| Leadership and Governance  Leadership and Governance  Leadership and Governance | **Governance** | Poor governance leading to increasing the cost of care |  |  |  |  |  |  |  |  |  |  |  |  |
|  | **Policy/strategy** | No state level uniform policy for relevant supportive policies, like discharge policy (medical colleges vs District level & peripheral SNCUs) |  |  |  |  |  |  |  |  | A |  |  |  |
|  |  | Strategy is available but not prioritized |  |  |  |  |  |  |  |  |  |  |  |  |
|  |  | No standard plan/policy for care of sick babies |  |  |  |  |  |  |  |  |  |  | A,G |  |
|  |  | Only physicians authorized for prescribing the drugs. ANMs skilled to provide only oxygen but not to prescribe |  |  |  |  |  |  |  |  | O,A |  | A |  |
|  |  | Management of sick newborns is not part of BPHS/EPHS package so service delivery does not exists |  |  |  |  |  |  |  |  |  |  |  |  |
|  | **Guidelines / standards** | No guidelines or recommendations |  |  |  |  |  |  |  |  |  |  | A, K, P |  |
|  |  | There is a WHO Guideline on inpatient care but not well circulated |  |  |  |  |  |  |  |  |  |  |  |  |
|  |  | Feeding is not emphasized in the standard treatment guidelines at district level |  |  |  |  |  |  |  |  |  |  |  |  |
|  |  | Guidelines do not promote family-centered care |  |  |  |  |  |  |  |  |  |  |  |  |
|  |  | Dissemination of guidelines is limited to senior officials, especially in tertiary care hospitals and does not reach the service providers at lower levels |  |  |  |  |  |  |  |  | A |  |  |  |
|  |  | Guidelines in place but not in practice (e.g. do not stay for 48 hours). Following the guidelines varies from institution to institution due to technical limitations |  |  |  |  |  |  |  |  | O |  |  |  |
|  | **Protocol** | Non-adherence to and lack of standard, clinical protocols for treatment of sick newborns and lack of adherence putting further burden upon institution and parents |  |  |  |  |  |  |  |  |  |  | A, G, K, P |  |
|  | **Public-private partnership** | Lack of clear public-private partnership |  |  |  |  |  |  |  |  | O |  |  |  |
|  | **Awareness** | Lack of awareness |  |  |  |  |  |  |  |  |  |  |  |  |
| Health Financing  Health Financing | **Funding** | Competing needs for available funds |  |  |  |  |  |  |  |  |  |  |  |  |
|  |  | Insufficient / lack of funds for distribution of necessary equipment and services (e.g. for lab support and blood components) |  |  |  |  |  |  |  |  | O |  | A, G, K |  |
|  |  | Availability of funds - not allocated for inpatient care for sick and small newborns at all recommended levels of care |  |  |  |  |  |  |  |  |  |  | K |  |
|  |  | Problems in funds disbursement at district / lower level and fund flow from national and state level |  |  |  |  |  |  |  |  | O, A |  |  |  |
|  |  | Low state subsidy |  |  |  |  |  |  |  |  |  |  |  |  |
|  | **Insurance** | Almost no insurance companies and lack of coverage of certain medicines for treatment of preterm newborns at certain levels |  |  |  |  |  |  |  |  |  |  |  |  |
|  | **Financial barriers to care** | Government cannot promote adequate medicine due to financial barriers |  |  |  |  |  |  |  |  |  |  | A, P |  |
|  |  | LBW babies need treatment, but financial problems are barriers to treat them |  |  |  |  |  |  |  |  |  |  | B |  |
|  |  | Hospital acquired infection requires costly antibiotics |  |  |  |  |  |  |  |  |  |  | A |  |
|  | **Out-of-pocket expenses / user fees** | No grants to families to pay for care |  |  |  |  |  |  |  |  |  |  |  |  |
|  |  | User fees - patient having to purchase drugs and out of pocket payment |  |  |  |  |  |  |  |  | A |  | K |  |
|  | **Awareness** | Clients and service providers not aware of free entitlements and available funds |  |  |  |  |  |  |  |  | O |  |  |  |
| Health Workforce  Health Workforce | **Number, distribution and role of health workers** | Lack of trained personnel in neonatal care in quantity and quality (knowledge, training and skills), particularly at lower levels |  |  |  |  |  |  |  |  | A |  | A, G, K, P |  |
|  |  | Disparity in the distribution of personnel (urban vs. rural) |  |  |  |  |  |  |  |  | O |  | P |  |
|  |  | Selection and posting of trainees (inappropriate selections and post-training deployment) |  |  |  |  |  |  |  |  | O |  |  |  |
|  |  | No integrated care for sick newborns by community workers |  |  |  |  |  |  |  |  |  |  |  |  |
|  | **Supervision** | Lack of supportive supervision |  |  |  |  |  |  |  |  | O |  |  |  |
|  | **Accountability** | No accountability |  |  |  |  |  |  |  |  |  |  | G |  |
|  | **Incentives and motivation** | No providers’ incentive scheme (e.g. lack of encouragement for task-shifting) leading to lack of motivation |  |  |  |  |  |  |  |  |  |  |  |  |
|  | **Training** | No/inadequate competency-based training/CME in care of small and sick babies (particularly at lower levels) including pre-and in-service and refresher training |  |  |  |  |  |  |  |  | O, A |  | A, G |  |
|  | **Guidelines and instructions** | Job descriptions available but not known/clear to all |  |  |  |  |  |  |  |  | O |  |  |  |
|  |  | No job chart available on the specific mentioned areas |  |  |  |  |  |  |  |  | A |  |  |  |
| Essential Medical Products and Technologies  Essential Medical Products and Technologies | **Lack of supplies and equipment** | General stock-outs / lack of supplies and equipment / inadequate supply |  |  |  |  |  |  |  |  | O, A |  | A,  G, P |  |
|  |  | Electronic weighing machine not available in all units |  |  |  |  |  |  |  |  | O |  |  |  |
|  |  | 0.45 NaCl/5% glucose not available |  |  |  |  |  |  |  |  | A |  |  |  |
|  |  | Shortfall in supply of oxygen due to demand and supply gap and non-availability |  |  |  |  |  |  |  |  | A |  | A, G |  |
|  |  | NICU in tertiary care and teaching hospitals in capital city has only ventilator support with oxygen |  |  |  |  |  |  |  |  |  |  | A |  |
|  | **Essential Medical List (EML)** | No oxygen in the list of essential features |  |  |  |  |  |  |  |  |  |  |  |  |
|  |  | All equipment required for oxygen are not included in the LNME (other than those included in the kit but not for continuity and many children) |  |  |  |  |  |  |  |  |  |  |  |  |
|  |  | Fluids not on list |  |  |  |  |  |  |  |  |  |  | P |  |
|  | **Policy** | The essential medicine list policy is available but not fully implemented |  |  |  |  |  |  |  |  | A |  |  |  |
|  |  | No national policy of supplies |  |  |  |  |  |  |  |  |  |  | G |  |
|  | **Procurement and supply management** | Poor and inaccurate forecasting, procurement, distribution and supply management (e.g. not available at all levels, no system in place to forecast demand for oxygen and IV fluids, issues with supply chain management and maintenance of supplies) |  |  |  |  |  |  |  |  | O, A |  | B, K |  |
| Health Service Delivery  Health Service Delivery  Health Service Delivery  Health Service Delivery | **Service availability / capacity of services** | Some facilities have limited availability of services (e.g. services for caring for low birth weight / small babies) |  |  |  |  |  |  |  |  | A |  | B |  |
|  |  | Side labs with special newborn care units not functioning optimally |  |  |  |  |  |  |  |  |  |  |  |  |
|  |  | Limited number of facilities providing inpatient care for severely sick and small newborns (particularly at lower levels) |  |  |  |  |  |  |  |  |  |  | K, P |  |
|  |  | Skewed distribution of Health facilities between rural and urban |  |  |  |  |  |  |  |  |  |  |  |  |
|  |  | No professional postnatal care system |  |  |  |  |  |  |  |  |  |  |  |  |
|  |  | Many available building plans are outdated for recommended inpatient care of sick and small baby |  |  |  |  |  |  |  |  |  |  |  |  |
|  |  | National scale-up of inpatient care for supportive care for sick and small/LBW yet to be done beyond selected hospitals supported by projects |  |  |  |  |  |  |  |  |  |  |  |  |
|  |  | No nursery and proper management of LBW babies |  |  |  |  |  |  |  |  |  |  | B |  |
|  | **Referrals** | Referrals are not as per the guidelines |  |  |  |  |  |  |  |  | O |  |  |  |
|  | **Space** | Limited space |  |  |  |  |  |  |  |  | O |  | A |  |
|  |  | No space for mothers of inpatient newborns to stay when their babies are admitted to facilities |  |  |  |  |  |  |  |  |  |  |  |  |
|  | **Quality improvement** | Poor adherence to existing guidelines/ recommendations/ protocols and no strategies in place for improvement |  |  |  |  |  |  |  |  |  |  |  |  |
|  |  | Lack of guidelines to improve the quality of services |  |  |  |  |  |  |  |  |  |  |  |  |
|  |  | No standardized quality tools and consequent performance reviews |  |  |  |  |  |  |  |  |  |  |  |  |
|  |  | Quality of care at facilities is a major issue / inadequate |  |  |  |  |  |  |  |  | O |  |  |  |
|  |  | Inadequate monitoring, quality assurance, maintenance and improvement mechanisms (including performance improvements) |  |  |  |  |  |  |  |  |  |  | K |  |
|  |  | Lack of mechanism to ensure the guideline are properly applied and adhered to |  |  |  |  |  |  |  |  |  |  |  |  |
|  |  | Mentoring / supervision guidelines not in place |  |  |  |  |  |  |  |  | A |  |  |  |
|  |  | Service quality is different between levels of care |  |  |  |  |  |  |  |  |  |  |  |  |
|  | **Health worker attitudes** | Poor health worker attitudes toward care of newborns |  |  |  |  |  |  |  |  | A, O |  |  |  |
|  | **Communication** | Ineffective communication (with clients and between community and facility) |  |  |  |  |  |  |  |  | A |  |  |  |
|  | **Supportive environment** | No family-centred, supportive care for the mother and newborn |  |  |  |  |  |  |  |  |  |  |  |  |
|  |  | Facilities lack supportive environment (e.g. for feeding) |  |  |  |  |  |  |  |  | O |  |  |  |
|  |  | Non-compliance with hygiene standards in the majority of structures |  |  |  |  |  |  |  |  |  |  |  |  |
| Health Information System  Health Information System  Health Information System | **Tools for information system** | Neonatal registers not developed |  |  |  |  |  |  |  |  |  |  |  |  |
|  |  | Records not being maintained uniformly at both higher and lower level facilities |  |  |  |  |  |  |  |  | A |  |  |  |
|  | **Indicators** | No harmonization of information on outcomes of hospital care in the registers |  |  |  |  |  |  |  |  |  |  |  |  |
|  |  | HMIS for inpatient supportive care for sick and small newborn is not yet functioning in all facilities or captures relevant information |  |  |  |  |  |  |  |  |  |  | G |  |
|  |  | Few indicators on newborns / no definition of indicators on sick newborns, only deaths |  |  |  |  |  |  |  |  |  |  |  |  |
|  |  | Lack of tracking of referrals and treatment |  |  |  |  |  |  |  |  | O |  |  |  |
|  |  | Lack of information on management system for sick newborns or LBW management |  |  |  |  |  |  |  |  |  |  | B, K |  |
|  | **Use and dissemination of information** | Poor dissemination and utilization of health information materials |  |  |  |  |  |  |  |  |  |  |  |  |
|  |  | SNCU software in place but not analysed regularly |  |  |  |  |  |  |  |  | O |  |  |  |
|  | **Quality improvement tools** | No extension sheets review of neonatal deaths |  |  |  |  |  |  |  |  |  |  |  |  |
|  |  | Standardized tools such as check lists for quality of inpatient care needs review to ensure optimal inpatient newborn care and proper dissemination |  |  |  |  |  |  |  |  |  |  |  |  |
|  | **Quality improvement assessment system** | Absence/poor coverage of clinical audits and perinatal deaths listed |  |  |  |  |  |  |  |  | O |  | A, B, P |  |
|  |  | No regular reviews on mortality and performance at hospital level and at district level |  |  |  |  |  |  |  |  | A |  | A |  |
|  |  | Technical information needed for treatment is not properly filled out |  |  |  |  |  |  |  |  |  |  |  |  |
|  |  | Data not disaggregated into specific neonatal causes |  |  |  |  |  |  |  |  |  |  |  |  |
| Community Ownership and Partnership  Community Ownership and Partnership  Community Ownership and Partnership | **Promotion / communication** | Communication materials only in French |  |  |  |  |  |  |  |  |  |  |  |  |
|  |  | No communication strategy in place |  |  |  |  |  |  |  |  |  |  |  |  |
|  |  | Lack of information on newborn care |  |  |  |  |  |  |  |  |  |  |  |  |
|  | **Awareness** | Lack of community awareness (e.g. of treatment process, care-seeking, available care and rights) |  |  |  |  |  |  |  |  | O |  | K, G |  |
|  |  | No specific efforts to increase the awareness of the general public, adolescent girls, pregnant women and young couples of the benefits of timely recognition of signs and early health seeking for sick newborns |  |  |  |  |  |  |  |  |  |  | B |  |
|  | **Care-seeking and referral** | Females have limited mobility (and say in decision-making) and males may not be available all the time that care should be sought |  |  |  |  |  |  |  |  | A |  |  |  |
|  |  | Poor health seeking behaviour in community |  |  |  |  |  |  |  |  | A |  |  |  |
|  |  | Poor referral system and linkages between facility and community |  |  |  |  |  |  |  |  | A |  | B |  |
|  | **Barriers / challenges faced by mothers** | Socio-cultural barriers |  |  |  |  |  |  |  |  | A |  |  |  |
|  |  | Delay in recognition of danger signs and prompt referral |  |  |  |  |  |  |  |  | A |  |  |  |
|  |  | Transport available but not utilized |  |  |  |  |  |  |  |  | O |  |  |  |
|  |  | Illiteracy |  |  |  |  |  |  |  |  |  |  |  |  |
|  |  | Loss of wages |  |  |  |  |  |  |  |  | O |  |  |  |
|  |  | Privacy |  |  |  |  |  |  |  |  | O |  |  |  |
|  |  | Communities suffer due to increasing costs of IV funds, antibiotics and other cost incurred |  |  |  |  |  |  |  |  |  |  | G |  |
|  |  | Lack of social security system for poor |  |  |  |  |  |  |  |  |  |  | G |  |
|  | **Access** | Inability to / limited access to services at all levels |  |  |  |  |  |  |  |  |  |  |  |  |
|  | **Community involvement and mobilization** | Poor involvement of men |  |  |  |  |  |  |  |  |  |  |  |  |
|  |  | Poor engagement of community |  |  |  |  |  |  |  |  |  |  | A,K,  P |  |
|  |  | Successful community mobilization models not available / limited community mobilization |  |  |  |  |  |  |  |  | O |  | S |  |

# B. Table S2: Solutions for inpatient care for small and sick newborns

| Inpatient Care for Sick and Small Newborns | | | | | | |
| --- | --- | --- | --- | --- | --- | --- |
| Health System Building Block | Africa | | | | | |
|  | Cameroon | DRC | Kenya | Malawi | Nigeria | Uganda |
| Leadership and Governance | - Revise the IMCI module will take better account of the newborn | - Strengthening of structures in equipment and organization of hospital care for small children / with low birth weight as recommended - Capacity building of staff in extra care to sick newborns and small / low birth weight - Extension of hygiene standards to the level of structures | - Building and reorganisation of facilities - Review the guideline/strategies and include medical social guidelines | - No solutions proposed | - Create enabling environment with targeted advocacy - Promote effective Coordination at all levels of care - Making guidelines widely available in Primary, secondary and tertiary facilities - Strengthening the quality assurance teams to monitor and supervise - Full dissemination of all relevant guidelines - PAN / NISONM to support the process | - No solutions proposed |
| Health Financing | - Advocacy for the mobilization of funds for the care of the newborn | - Advocacy to increase government subsidy - Creation and expansion of mutual health | - Increase resource mobilisation and prioritise needs - Alternative funding mechanism | - No solutions proposed | - Health insurance for out of pocket payment with emphasis on community based health insurance - Strengthen logistics management system (appropriate forecasting, quantification based on periodic needs assessment) | - No solutions proposed |
| Health Workforce | - Initial and continuing training of health personnel in the care of the newborn | - Integration of competency-based training at the initial training - Policy affection balanced staff - Capacity building of staff - Extension of the FBR | - Recruitment and redistribution - Scale up the in-service training and update pre-service training | - No solutions proposed | - Mapping of health worker - Remuneration and Motivation for rural posting - Fast track the implementation of task shifting policy - Strengthen / support periodic competence based training (mandatory CMEs of categories of health workers) | - No solutions proposed |
| Essential Medical Products and Technologies | - Plea to include oxygen on the essential drug list - Equipping health facilities with oxygen extractors - Improve inventory management | - Update the LNME - Advocacy for increased funding and strengthening of SNAME | - Capacity building of personnel and streamline the procurement | - No solutions proposed | - Implementation of essential medicine policy to ensure enlisted commodities and medicines are available and accessible at service delivery points - Strengthening of logistic management systems - Ensure Abuja declaration of 15% allocation for health budget | - No solutions proposed |
| Health Service Delivery | - See above | - Extension of therapeutic protocols - Advocate for funds supervision - FBR | - Update recommended government building plans - Increase the number of health facilities | - No solutions proposed | - Decentralization of in- patient neonatal care: Sick but Not critically ill newborns can be managed at primary health and community settings (home based care) - Mother friendly neonatal in - patient facilities should be encouraged | - No solutions proposed |
| Health Information System | - See above | - Definition of indicators for newborn patients and their integration in the NHIS - Extension sheets reviewing neonatal deaths - Extension and auditing of perinatal deaths | - Develop neonatal registers - Ensure data is disaggregated | - No solutions proposed | - Support dissemination and utilization of health information materials | - No solutions proposed |
| Community Ownership and Partnership | - No solutions proposed | - Translation of MNCH communication materials in local languages - Increased awareness for men's involvement - Creation and expansion of mutual health - Implementation of OAC for women's literacy | - Health promotion - Community involvement | - No solutions proposed | - Promotion of Male involvement in newborn care including KMC - Increase the public enlightenment on sick newborn signs and importance of early health seeking for the sick newborn | - No solutions proposed |

| Inpatient Care for Sick and Small Newborns | | | | | | |
| --- | --- | --- | --- | --- | --- | --- |
| Health System Building Block | Asia | | | | | |
|  | Afghanistan | Bangladesh | India - AP | India - Odisha | Nepal | Vietnam |
| Leadership and Governance | - No proposed solutions | - Advocacy with policymakers and development partners and policy dialogue for upgrading hospitals/facilities with provision of inpatient care for supportive care for sick and small/LBW babies - Adequate budget allocation in Ops of DGHS and DGFP for provision of inpatient supportive care for sick and small/LBW babies - Introduce effective supervision and monitoring system | - Ensure effective dissemination for the new guidelines (Workshop, website which is regularly updated for service providers) - Monitoring/Audit to ensure dissemination and implementation of guidelines - Uniform guidelines to be framed with support of professional bodies (NNF, IAP, FOGSI) | - Referrals sheets to be plotted, baby should be stabilized before referrals, and to be accompanied by trained personnel, day off from the work should be given - Establishing High priority regional health centres - Few ANMs to be trained on administering IV antibiotics on discharge at home - Implementation needs to be strengthened | - Revision of the strategy to prioritize the sick babies during postpartum period - Develop clinical guideline in line with revised strategy | - To develop and approve the guidelines |
| Health Financing | - No proposed solutions | - Allocation of adequate budget in the operational plans of DGHS and DGFP for upgrading facilities with provision of inpatient care for sick and small newborn | - IT enabled mechanisms to expedite the process of fund disbursement SNCUI/Cs to participate in the PIP process, and given responsibility for contingency fund – 25% to 30% for day to day management - IEC campaign at facility and community level to increase awareness about entitlements-JSSK - Financial support for operationalizing lab and blood bank facilities | - Fund flow should be streamlined with time frame - Medical colleges should be included in free treatment category - Loss of wages compensation to the attendant including diet, rest shed with toilet facilities | - Abolishment of user and drugs fees - Safe Newborn Care incentive scheme | - To apply health insurance at all levels for sick and small/LBW newborns - To ensure adequate budget for procurement of medicine and equipment for all levels |
| Health Workforce | - No proposed solutions | - Adequate budget allocation in OPs for training and refresher training of service providers | - IT enabled training database linked to HR profile, placement - Refresher trainings, supportive supervision, mentoring by the Medical colleges & Pvt institutions - ANMs and Staff Nurses to be empowered for injectable antibiotics - Recruitment and training of local staff, hard area allowances and performance based incentives to improve retention - Job description of all cadres to be made available at all levels of facilities | - Job chart should be in place - Guidelines to be prepared and staff to be trained - Accreditation of private Nursing colleges (by ONC) - ANM Needs to be trained in IV fluids and IGT feeding - Nurses/ MOs/Specialist to be trained on FIMNCI - Refresher training for Health workers - Skill lab hands-on training is essential | - Revise the JDs of ANM, staff nurse, AHW and HA to provide them authority for prescription of IV fluids | - To deploy adequately for lower levels, especially for remote areas - To develop plan for training and certification on care and treatment for newborns |
| Essential Medical Products and Technologies | - No proposed solutions | - Introduce Quality Assurance at facilities providing inpatient newborn care | - Supply chain management to be made effective at all levels using IT - Training of facility managers on generating demand for medicines as per need - Facility assessments of those reporting frequent stock-outs | - Forecast based on data and Buffer to be kept - 0.45 NaCl/5% glucose should be supplied - Provision of AMC/CMC, Supply chain management and provision of central oxygen to be enhanced and continued - Special equipment like, cpap, portable x-ray, USG and abg analyser provision should made for tertiary care centres - Supply of simple drugs like Paracetamol and Antibiotics | - Incorporate the forecasting, procurement, distribution into the central as well as decentralized procurement system - Develop the hospital based LMIS system for ensuring adequate stock level - Automation in the stores to prevent disposal and waste | - To supply adequately equipment, medicines, transfusion solutions according to the need for treatment - To strengthen the system |
| Health Service Delivery | - No proposed solutions | - Allocation of budget in OPs to ensure supply of equipment and logistics for management of inpatient sick and small newborn has to be ensured | - Quality Assurance cell at the state level with regular quality assessments (emphasis on supportive supervision & mentoring by Medical colleges/Pvt hospitals) - Evidence informed IEC/BCC strategy; IPC skill enhancement for health care providers - Two way referral communication to be established | - Increase in bed strength, food for attendants, facility to stay - Space for step down area should be increased - Scope for PPP mode - BCC to Service providers | - Develop quality improvement strategy (CME, audit, reminders) for improving the adherence to clinical standards | - To improve quality of health services in terms of equipment and human resources - To organize professional training on care for sick and small/LBW newborns |
| Health Information System | - No proposed solutions | - Introduce functioning HMIS for inpatient supportive care for sick and small newborn in all facilities; national scale up of clinical audits and perinatal death reviews | - Software apps for record keeping and establishing linkages from facility to community (follow up of SNCU graduates thru ANMs) - Regular review meetings on performance, survival statistics with MO I/C in the lead, and oversight from higher state officials - List of dashboard indicators to be established | - Introduction of CDR and Still birth audits - Software for other newborns to be introduced - Provision of MCTS in case sheet - Regular mentoring and PPP mode | - Develop a checklist to record the information on sick newborns or LBW management - Integrate the recording of management of sick newborns and LBW babies into the HMIS | - To apply thorough statistic and reporting system for sick/LBW newborns - To develop concise, rational, informative clinical record forms for sick and small/LBW newborns |
| Community Ownership and Partnership | - No proposed solutions | - Appropriate IEC materials need to be developed to use for BCC - Establish effective referral system with involvement of community group/community support groups | - IEC/BCC for community awareness and for engaging male members of the family in the care - Ensure linkages between facility and community - Post discharge counselling on danger signs and referral mechanisms to be strengthened - Women Empowerment | - IEC/BCC on hand wash and personal hygiene - MHU to be sensitized & trained - Studies to be explored - Community awareness on entitlement of programmes to be strengthened | - Develop the communication strategy, plan, tools and messages to improve care seeking and care taking practices | - To strengthen IEC/BCC for improving access of community to newborn care services (via mass media, prenatal counselling) |

| Inpatient Care for Sick and Small Newborns |
| --- |

| Health System Building Block | Pakistan | | | | | |
| --- | --- | --- | --- | --- | --- | --- |
|  | AJK | Baluchistan | Gilgit-Baltistan | Khayber Pakhtun | Punjab | Sindh |
| Leadership and Governance | - Standard protocols to be developed for severely sick newborn | - No solutions proposed | - Prioritization of issue - Introduction of standard protocols | - Develop, adopt and implement National Guidelines on Full supportive care for severely sick and small newborn | - Guidelines or policies should be developed for DHQs and above | - No solutions proposed |
| Health Financing | - Increase allocation of funds - Seek alternate funding /donor support - Public private partnership | - No solutions proposed | - Rational allocations - Budget increasing | - Enhance budgetary allocations | - Financial improvement in government and private sector | - No solutions proposed |
| Health Workforce | - Increase number of health workers - Training program to enhance their competencies - Periodic monitoring visits to check their competencies & adherence to standards | - No solutions proposed | - Adherence to clinical protocols and accountability | - Have sufficient and well trained workforce to provide inpatient care | - Training needs to be scaled up | - No solutions proposed |
| Inpatient Care for Sick and Small Newborns | | | | | | |
| Health System Building Block | **Pakistan** | | | | | |
|  | AJK | Baluchistan | Gilgit-Baltistan | Khayber Pakhtun | Punjab | Sindh |
| Essential Medical Products and Technologies | - Constant supply needs to be ensured | - No solutions proposed | - Lack of priorities - Lack of O2 supplies - Antibiotic purchase according standard protocols - Purchase of oxygen concentrator | - No solutions proposed | - Fluids need to be added to NEM - Constant supply ensured | - No solutions proposed |
| Health Service Delivery | - National standard clinical protocols should be available and ensured at all levels | - No solutions proposed | - No solutions proposed | - Enhance the coverage of services based on geographic needs | - Should be added to the district level hospitals portfolio | - No solutions proposed |
| Health Information System | - An integrated policy should be formulated and implemented in tertiary care hospitals | - No solutions proposed | - No solutions proposed | - Make this information as integral part of HMIS | - An integrated policy should be formulated | - No solutions proposed |
| Community Ownership and Partnership | - Community participation and knowledge should be improved | - No solutions proposed | - No solutions proposed | - No solutions proposed | - Community participation and knowledge should be improved | - No solutions proposed |

# C. Figure S1: Subnational grading of bottlenecks for inpatient care of small and sick newborns

# D. Literature Search Strategy

**For the background section, we used the following search terms in Pub Med. Limits were applied and only relevant articles were retrieved.**

**Newborn**

(neonat* OR newborn* OR new-born* OR infant, newborn/ OR infant, low birth weight/ OR infant, small for gestational age/ OR infant, very low birth weight/ OR infant, extremely low birth weight/ OR infant, premature/ OR infant, extremely premature/)

AND

**Oxygen**

(Oxygen therapy OR o2 therapy OR continuous positive airway pressure OR CPAP or nasal prong or nasal cannula* OR head box or (oxygen or o2))

AND

**Enteral/intragastric feeding**

(Nasogastric feed* OR orogastric feed* OR oro-gastric feed* OR naso-gastric feed* OR enteral feed* OR enteral nutrition OR gavage feed*)

AND

**IV fluids**

(Maintenance fluid* OR IV fluid* OR intravenous fluid* OR supplemental fluid* OR infusion*)

**For the discussion section we searched the following terms in pub med and google. Only relevant articles were retrieved.**

**Health Financing**

(Health) AND (financial access OR financial barrier OR out-of-pocket payment OR user fees OR conditional cash transfers OR cash benefits OR performance based incentives OR voucher OR reimbursement of transport costs)

Budget allocation

Innovative funding

Social health insurance

Universal health insurance

Community based insurance

National health insurance

**Health workforce**

(Heath worker OR staff) AND (pre-service training OR in-service training OR recruitment OR recognition of midwifery staff OR task-shifting OR training OR performance incentive OR retention OR contracting out OR increase in availability OR skill mix OR remuneration OR salaries)

Community health workers

Task shifting

Skills based training

Neonatal nursing

Competency based training

**Community:**

(Health) AND (information education communication OR community mobilization OR utilization OR sensitization OR male involvement)

(Health) AND (education) AND (leaders OR women)

Community awareness

Linkages

Care-seeking

Women’s group

Male involvement

Behavior change communication (BCC)

Community and facility linkages

Community engagement

# E. Figure S2: Overcoming bottlenecks for the management of neonatal jaundice: diagnostics and devices

| **Overcoming bottlenecks for the management of neonatal jaundice: diagnostics and devices** |
| --- |
| Neonatal hyperbilirubinaemia is common, whilst most mild cases resolve spontaneously, failure to recognise and institute timely effective treatment for potentially severe disease can lead to bilirubin induced brain damage (kernicterus) and neonatal death. A systems-based approach is crucial to prevent adverse outcomes[[1](#_ENREF_1)]. This will require innovative strategies and affordable technologies to bridge the existing social and access barriers in micro and macro-health environment [[2](#_ENREF_2)] which may include: |
| ***Improved identification and management of underlying causes:*** Rh hemolytic disease is an important established preventable cause of kernicterus, point-of-care Rh blood typing, minimally at the time of birth with unfettered targeted access to Rh immunoprophylaxis is critical for prevention. There is need for a panel of bilirubin tests for hemolysis and glucose-6-phoshate dehydrogenase deficiency. |
| ***Overcoming Barriers to diagnosis:*** Icterometry is a low cost, simple, effective diagnostic tool. Coupled with jaundice education as part of postnatal care delivered by rural health-care workers it improved care-seeking and a reduced bilirubin levels in a Vietnamese cluster-randomized study[[3](#_ENREF_3)]. In a community birthing facility in Brazil Screening, using an Ingram icterometer or transcutaneous bilirubin during the first day after birth, with promotion of breastfeeding and timely use of phototherapy was associated with very low (0.82%) readmission rates with none requiring exchange transfusions[[4](#_ENREF_4)]. |
| ***Overcoming Intervention Barriers:*** Effective phototherapy implies its use as a “drug” at specific blue light wavelengths, emission spectrum in a precise (narrow) bandwidth to up to 80% of an infant’s body surface. The efficacy of longer-lasting LED lights that meet clinical and engineering expectations have been demonstrated in low and middle income settings [[5](#_ENREF_5),[6](#_ENREF_6)]. These devices are also the most affordable and effective for at least one year of continuous use and are poised for implementation at both primary and secondary birthing facilities.  **Plans and Challenges** are not just limited to development of novel screening and prevention technologies, improving access to healthcare or monitoring global benchmarks of unacceptable neonatal morbidities. These need to be embedded in healthcare systems that are accountable to the mother and her family. |

# F. Table S3: Significant bottlenecks and solution themes by health systems building block

| **Health system building block** | **Subcategories** | **Significant bottlenecks** | **# of countries** | **Selected solution (themes)** |
| --- | --- | --- | --- | --- |
| **Leadership and Governance** | Policy/Strategy | - Management of sick newborns is not part of the BPHS/EPHS package so service delivery does not exist (CAMEROON, AFGHANISTAN) | 2 | *Advocacy and political will and improve organization structures* |
|  |  | - No guidelines or recommendations (NIGERIA, NEPAL, PAKISTAN, VIETNAM) | 4 | *Review and disseminate guidelines* |
|  | Guidelines/standards | - Guidelines in place but not in practice (e.g. do not stay for 48 hours). Following the guidelines varies from institution to institution due to technical limitations (INDIA, VIETNAM) | 2 | *Review and disseminate guidelines* |
|  |  | - Guidelines do not promote family-centered care (KENYA, VIETNAM) | 2 | *Review and disseminate guidelines* |
| **Health Financing** | Funding | - Insufficient/lack of fund for distribution of necessary equipment and services (e.g. for lab support and blood components) (CAMEROON, MALAWI, INDIA, PAKISTAN) | 4 | *Budget allocation* |
|  |  | - Availability of funds – not allocated for inpatient care for sick and small newborns at all recommended levels of care (PAKISTAN, AFGHANISTAN, BANGLADESH) | 3 | *Budget allocation* |
|  | Public Private Partnership | - Lack of clear public-private partnership (CAMEROON, INDIA) | 2 | *Innovative funding mechanisms* |
|  | Insurance and out of pocket expenses | - Almost no insurance companies and lack of coverage of certain medicine for treatment of preterm newborns at certain levels (DRC, VIETNAM) | 2 | *Innovative funding mechanisms* |
| **Health system building block** | **Subcategories** | **Significant bottlenecks** | **# of countries** | **Selected solution (themes)** |
|  |  | - User fees – patient has to purchase drugs and out of pocket payments and no grants exist to families to pay for care (KENYA, NIGERIA, INDIA, NEPAL, PAKISTAN, VIETNAM, CAMEROON) | 7 | *Innovative funding mechanisms* |
| **Health Workforce** | Number, distribution and role of health workers | - Lack of trained personnel in neonatal care in quantity and quality (knowledge, training, skills), particularly at lower levels (CAMEROON, DRC, KENYA, MALAWI, BANGLADESH, INDIA, PAKISTAN, VIETNAM) | 8 | *Competency based training* |
|  |  | - Only physicians authorized for prescribing the drugs. ANMs skilled to provide only oxygen but not to prescribe. (INDIA, NEPAL, PAKISTAN) | 3 | *Task-shifting* |
|  |  | - Disparity in the distribution of personnel (urban vs rural) (CAMEROON, DRC, NIGERIA, INDIA, PAKISTAN) | 5 | *Innovative recruitment and retention strategies* |
|  | Supervision | - Lack of supportive supervision (CAMEROON, DRC, NIGERIA, BANGLADESH, INDIA) | 5 | *Innovative recruitment and retention strategies* |
|  | Incentives and motivation | - No providers incentive scheme (DRC, NIGERIA, NEPAL) | 3 | *Innovative recruitment and retention strategies* |
|  | Training | - No/inadequate competency based training/CME in care of small and sick babies (particularly at lower levels) including pre and in-service and refresher training (DRC, NIGERIA, AFGHANISTAN, INDIA, NEPAL, PAKISTAN, VIETNAM) | 7 | *Competency based training* |
|  | Guidelines and Instructions | - Job descriptions available but not known/clear to all (CAMEROON, INDIA) | 2 | *Innovative recruitment and retention strategies* |
| **Health system building block** | **Subcategories** | **Significant bottlenecks** | **# of countries** | **Selected solution (themes)** |
| **Essential Medical Products and Technologies** | Lack of supplies and equipment | - General stock outs/lack of supplies and equipment/inadequate supply (DRC, MALAWI, AFGHANISTAN, BANGLADESH, INDIA, PAKISTAN, VIETNAM) | 7 | *Logistic system strengthening* |
|  |  | - Shortfall in supply of oxygen due to demand and supply gap and non-availability (INDIA, PAKISTAN) | 2 | *Updating essential medicine list* |
|  | Policy | - The essential medicine list is available but not fully implemented. (NIGERIA, INDIA) | 2 | *Updating essential medicine list* |
|  | Procurement and supply management | - Poor and inaccurate forecasting procurement distribution and supply management (e.g. not available at all levels, no system in place to forecast demand for oxygen and IV fluids, issues with supply chain management and maintenance supplies) (KENYA, NIGERIA, BANGLADESH, INDIA, NEPAL, PAKISTAN, VIETNAM) | 7 | *Forecasting*  *Logistic system strengthening* |
| **Health service delivery** | Service availability/capacity of services | - Some facilities have limited availability of services (eg services for caring for low birth weight/small babies) (CAMEROON, DRC, BANGLADESH, INDIA, PAKISTAN, VIETNAM) | 6 | *Increase service availability and rationalize service distribution* |
|  |  | - Limited number of facilities providing inpatient care for severely sick and small newborns (particularly at the lower level). (KENYA, PAKISTAN) | 2 | *Increase service availability and rationalize service distribution* |
|  | Space | - Limited space (MALAWI, INDIA, PAKISTAN, VIETNAM) | 4 | *Increase service availability and rationalize service distribution* |
|  | Quality improvement | - Inadequate monitoring, quality assurance, maintenance and improvement mechanisms (including performance improvements) (NIGERIA, BANGLADESH, PAKISTAN) | 3 | *Quality improvement and assurance (supervision, mentoring and quality assurance)* |
| **Health system building block** | **Subcategories** | **Significant bottlenecks** | **# of countries** | **Selected solution (themes)** |
|  |  | - Poor adherence to existing guidelines/recommendations/protocols and no strategies in place for improvement (CAMEROON, NIGERIA, NEPAL) | 3 | *Quality improvement and assurance (supervision, mentoring and quality assurance)* |
|  |  | - Lack of mechanism to ensure the guidelines are properly applied and adhered to (INDIA, VIETNAM) | 2 | *Quality improvement and assurance (supervision, mentoring and quality assurance)* |
|  |  | - Quality of care at facilities is a major issue/ inadequate (BANGLADESH, INDIA) | 2 | *Quality improvement and assurance (supervision, mentoring and quality assurance)* |
|  | Health worker attitudes | - No family-centred, supportive care for the mother and newborn (DRC, VIETNAM) | 2 | *Quality improvement and assurance (supervision, mentoring and quality assurance)* |
|  |  | - Poor health worker attitudes toward care of newborns (KENYA, INDIA) | 2 | *Improve working conditions* |
|  | Supportive environment | - Facilities lack supportive environment (e.g. for feeding) (KENYA, INDIA) | 2 | *Improve working conditions* |
| **Health Information System** | Indicators | - HMIS for inpatient supportive care for sick and small newborn is not yet functioning in all facilities or captures all relevant information (BANGLADESH, PAKISTAN, VIETNAM) | 3 | *Strengthening and integration of HMIS* |
| **Health system building block** | **Subcategories** | **Significant bottlenecks** | **# of countries** | **Selected solution (themes)** |
|  |  | - Few indicators on newborns/no definition of indicators on sick newborns, only deaths (DRC, VIETNAM) | 2 | *Develop indicator definitions, reporting systems and tools* |
|  |  | - Lack of information on management system for sick newborns or LBW management (NEPAL, PAKISTAN) | 2 | *Strengthening and integration of HMIS* |
|  | Quality improvement assessment system | - Absence /poor coverage of clinical audits and perinatal deaths listed (CAMEROON, DRC, MALAWI, AFGHANISTAN, BANGLADESH) | 5 | *Scale up audits and registers* |
|  |  | - No regular reviews on mortality and performance at hospital level and at district level (INDIA, PAKISTAN, VIETNAM) | 3 | *Scale up audits and registers* |
| **Community Ownership and Participation** | Awareness | - No specific efforts to increase the awareness of the general public, adolescent girls, pregnant women and young couples of the benefits of timely recognition of signs and early health seeking for sick newborns (NIGERIA, PAKISTAN) | 2 | *Raise community awareness* |
|  |  | - Lack of community awareness (eg of treatment process care-seeing available care and rights) (CAMAROON, DRC, AFGHANISTAN, BANGLADESH, INDIA, PAKISTAN) | 6 | *Accessibility of information and information and improve care seeking and linkages* |
|  | Care-seeking and referral | - Females have limited mobility (and say in decision-making) and males may not be available all the time that care should be sought (AFGHANISTAN, INDIA) | 2 | *Male involvement* |
|  |  | - Poor referral system and linkages between facility and community (INDIA, PAKISTAN) | 2 | *Improve linkages and care-seeking* |
| **Health system building block** | **Subcategories** | **Significant bottlenecks** | **# of countries** | **Selected solution (themes)** |
|  | Barriers/challenges faced by mothers | - Socio-cultural barriers (CAMEROON, KENYA, INDIA) | 3 | *Raise community awareness* |
|  | Access | - Inability to/limited access to services at all levels (CAMEROON, AFGHANISTAN) | 2 | *Accessibility of information and information and improve care seeking and linkages* |
|  | Community involvement and mobilisation | - Successful community mobilisation models not available/limited community mobilisation (INDIA, PAKISTAN) | 2 | *Raise community awareness* |
|  |  | - Poor engagement of community (DRC, PAKISTAN, VIETNAM) - Poor involvement of men (CAMEROON, DRC, KENYA, NIGERIA, INDIA, VIETNAM) | 3  6 | *Raise community awareness*  *Accessibility of information and information and improve care seeking and linkages*  *Male involvement* |

# G. References

1. Bhutani VK, Maisels MJ, Stark AR, Buonocore G (2008) Management of jaundice and prevention of severe neonatal hyperbilirubinemia in infants >or=35 weeks gestation. Neonatology 94: 63-67.

2. Bhutani VK, Zipursky A, Blencowe H, Khanna R, Sgro M, et al. (2013) Neonatal hyperbilirubinemia and Rhesus disease of the newborn: incidence and impairment estimates for 2010 at regional and global levels. Pediatr Res 74 Suppl 1: 86-100.

3. Luu MN, Le LT, Tran BH, Duong TK, Nguyen HT, et al. (2014) Home-use icterometry in neonatal hyperbilirubinaemia: Cluster-randomised controlled trial in Vietnam. Journal of paediatrics and child health 50: 674-679.

4. Facchini FP, Mezzacappa MA, Rosa IR, Mezzacappa Filho F, Aranha-Netto A, et al. (2007) Follow-up of neonatal jaundice in term and late premature newborns. Jornal de pediatria 83: 313-322.

5. Kumar P, Chawla D, Deorari A (2011) Light-emitting diode phototherapy for unconjugated hyperbilirubinaemia in neonates. Cochrane database of systematic reviews: CD007969.

6. Slusher TM, Zipursky A, Bhutani VK (2011) A global need for affordable neonatal jaundice technologies.
